# *Anchusa azurea* enhances cisplatin efficacy in oral and bone cancers through IL-17 and TNF-α pathway modulation: a metabolomic and network pharmacology approach

**DOI:** 10.1038/s41598-026-56489-3

**Published:** 2026-06-13

**Authors:** Sally A. Fahim, Alaadin E. El-Haddad, Rehab I. Moustafa, Shimaa O. Ali, Marwa Sharaky, Nouran H. El Sherazy, Aliaa A. Elsherbiny, Ashrakat Y. Nabawy, Ahmed A. Elwakil, Heba Alah Esam, Salma Emad, Menatallah Abdelrahman, Mohamad Ayman, Hind A. H. Soliman, Eman M. El-Deeb, Amr M. Saadeldeen

**Affiliations:** 1 Department of Biochemistry, School of Pharmacy, Newgiza University (NGU), Newgiza, km 22 Cairo-Alexandria Desert Road, Giza, 12577 Egypt; 2 Department of Pharmacognosy, Faculty of Pharmacy, October 6 University, Giza, 12585 Egypt; 3 Microbial Biotechnology Department, Biotechnology Research Institute , National Research Centre, Dokki, P.O. Box 12622, Cairo, Egypt; 4 Department of Microbiology, School of Pharmacy, Newgiza University (NGU), , Newgiza, km 22 Cairo-Alexandria Desert Road, Giza, 12577 Egypt; 5 Department of Biochemistry, Faculty of Pharmacy, Cairo University, Cairo, 11562 Egypt; 6 Cancer Biology Department, National Cancer Institute, Cairo University, Cairo, Egypt; 7 Department of Clinical Pharmacy, School of Pharmacy, Newgiza University (NGU), Km 22 Cairo-Alexandria Desert Road, P.O. Box 12577, Newgiza, Giza, Egypt; 8 Department of Clinical pharmacy, Cairo University Hospitals, Cairo university, Cairo, Egypt; 9 School of Pharmacy, Newgiza University (NGU), Newgiza, km 22 Cairo- Alexandria Desert Road, Giza, 12577 Egypt; 10 Pharmacognosy Department, Faculty of Pharmacy and drug technology, Egyptian Chinese University, Cairo, 11771 Egypt; 11 Department of Pharmacognosy, School of Pharmacy, Newgiza University (NGU), Newgiza, km 22 Cairo-Alexandria Desert Road, Giza, 12577 Egypt

**Keywords:** *Anchusa azurea*, Cisplatin, Synergistic effect, Oral cancer, Bone cancer, Network pharmacology, Biochemistry, Cancer, Drug discovery

## Abstract

*Anchusa* species have traditionally been used to treat arthritis, gout, rheumatism, and skin wounds. Cisplatin (Cis) is a widely used chemotherapy drug associated with serious adverse effects. The study aimed to evaluate the potential synergistic anticancer effects of *Anchusa azurea* methanol extract (AAME) in combination with cisplatin against bone, skin, and oral cancer cell lines. This study involved a comprehensive metabolomic profiling of AAME, alongside cytotoxicity assays, cell cycle analysis, autophagy assessment, and evaluation of IL-17 and TNF-α pathway-related protein expression. AAME inhibited the proliferation of MG63 and HNO97 cancer cells while sparing HSF normal cells. AAME and Cis displayed synergistic effects (combination index < 1), especially in HNO97 cells. Treatments led to a synergistic decrease in TNF-α, p/t-JNK, IL-17, pNFκB/tNFκB, TRAF6, pMAPK/tMAPK ratios, and AP1 expression, also increased Casp3 and Casp8 levels, cell cycle arrest, and enhanced autophagy. The TPC and TFC of AAME are 5.46 mgGAE/gE and 0.13 mgRE/gE respectively, reflecting on its radical scavenging activity (EC50 209.67 *µ*g/mL). HRLC-MS/MS leading to the annotation of 50 metabolites, including phenolics and flavonoid derivatives, notably with a prevalence of rosmarinic acid, quercetin, and kaempferol. In network pharmacology, the 90 genes are common between AAME constituents and oral cancer. *A. azurea* enhances cisplatin’s anticancer effects by modulating IL-17, TNF-α, and apoptotic pathways, offering a promising adjuvant therapeutic strategy. Further in vivo investigations are warranted to validate the observed in vitro synergistic anticancer effects of *A. azurea* in combination with Cis.

**Supplementary Information:**

The online version contains supplementary material available at 10.1038/s41598-026-56489-3.

##  Introduction

*Anchusa azurea* Mill. (syn. *Anchusa italica* Retz), a flowering plant belonging to the Boraginaceae family growing in North Africa, Europe, and Western Asia^1^. *A. azurea* has been employed for its stimulant, diuretic, demulcent, cathartic, and tonic effects^2^. Leaves’ decoction was used externally for wounds and to manage chest pain, colds, and sore throats^3^. Several studies have highlighted the pharmacological properties of *A. azurea*, including antibacterial, antioxidant, anti-inflammatory, and anticancer activities^1^. The hypouricemic activity of *A. azurea* was also observed, as it showed inhibitory effects on xanthine oxidase and significant lowering effects on serum liver creatinine and urea levels in mice^4^. These therapeutic effects are attributed to the diverse phytochemical profile of *A. azurea*, which includes phenolic acids, flavonoids, triterpene saponins, alkaloids, and fatty acids^5^. The flavonoid and triterpenoid contents have shown cardioprotective effects, particularly in models of myocardial infarction^6^. Rosmarinic acid isolated from the aerial parts of *A. azurea*, was found to have anti-inflammatory effects^7^. Furthermore, its antioxidant capacity has been confirmed through assays such as DPPH and ABTS, revealing concentration-dependent free radical scavenging activity^8^. *A. azurea* extracts have demonstrated inhibitory effects against various strains of microbes, including *Escherichia coli*, *Klebsiella pneumoniae*, coagulase-negative *Staphylococcus* species, and *Candida albicans*^8^. The presence of phenolics like chlorogenic and caffeic acids in *A. azurea* underscores its potential as an anticancer and antimicrobial agent^2^. Despite these reported biological activities, current knowledge remains limited regarding the comprehensive characterization of its bioactive metabolites and, more importantly, the mechanistic understanding of its anticancer effects, particularly in combination with conventional chemotherapeutic agents. In addition, the potential of *A. azurea* as a chemosensitizing agent and its impact on inflammation-driven oncogenic pathways have not been systematically investigated.

Cancer remains a major global health challenge and a leading cause of morbidity and mortality worldwide. According to recent global estimates, approximately 20 million new cancer cases and nearly 10 million cancer-related deaths were reported in 2022, highlighting the growing burden of the disease^9^. Oral cancer represents a significant subset, particularly in developing countries, where it is associated with high incidence and mortality rates due to late diagnosis and limited therapeutic options^10^. In parallel, osteosarcoma, although relatively rare, is the most common primary malignant bone tumor in children and young adults and is characterized by aggressive behavior and poor prognosis in metastatic cases^11^. These statistics underscore the urgent need for developing more effective and safer therapeutic strategies, including combination approaches that enhance the efficacy of conventional chemotherapeutics such as Cisplatin while minimizing their adverse effects. The anticancer potential of *A. azurea* has been supported by in vitro data showing selective cytotoxicity against HepG2, MCF-7, RKO, and A549 cell lines, while exhibiting low toxicity towards normal cell lines such as MCF-10 A and 3T3-L1^2^. However, these studies primarily focused on cytotoxicity without exploring the underlying molecular mechanisms or pathway-specific effects. Importantly, inflammation-associated signaling pathways, particularly IL-17 and TNF-α, play critical roles in tumor progression, survival, and metastasis in oral cancer, making them relevant therapeutic targets. Nevertheless, the potential modulation of these pathways by *A. azurea*, either alone or in combination with cisplatin (Cis), has not been previously elucidated.

^12–16^ Natural products are key sources of anticancer agents with low side effects, influencing biological pathways like cell death, proliferation, migration, angiogenesis, and metastasis, and triggering signals that lead to cancer cell death^17,18^. The combination of chemotherapeutic drugs and natural compounds, as *A. azurea*, is gradually becoming an important strategy for tumor treatment^19^. Their roles as chemosensitizers, immune therapeutics, and in combinatorial therapies enhance treatment efficacy and safety^20^. Therefore, the novelty of the present study lies in the integration of comprehensive metabolomic profiling with functional in vitro validation and network pharmacology analysis to investigate the synergistic anticancer effects of *A. azurea* methanol extract (AAME) in combination with Cis. This study specifically aims to elucidate the potential modulation of IL-17 and TNF-α signaling pathways, providing mechanistic insight into the observed effects. In addition, antimicrobial activity was assessed as part of a broader biological characterization of the *A. azurea* extract. Accordingly, this work not only evaluates the cytotoxic and synergistic effects of AAME in oral and bone cancer models but also explores its multi-target interactions through computational approaches, thereby offering a more comprehensive understanding of its potential as a natural adjuvant in cancer therapy.

##  Materials and methods

### Chemicals and reagents

Chemicals; aluminum chloride (HPLC Pvt. Ltd., India), dimethyl sulfoxide (SRL Chem, India), gallic acid (99%, Qualikems, India), rutin trihydrate (98%, Loba Chemie, India), and sodium nitrate (ADVENT, India) were of the highest grade. Folin–Ciocalteu and DPPH were obtained from Sigma-Aldrich, Germany. Other reagents and chemicals were purchased with the highest grade (PioChem, Egypt).

### Plant materials and extraction procedure

The dried aerial parts of *A. azurea* Mill. were purchased from the local herbal store, Egypt (Feb. 2024). It was identified in the Flora and Phyto-Taxonomy Research Centre, Agricultural Research Centre, Cairo, Egypt. A voucher sample (AZ/2024) was deposited at the herbarium of the Pharmacognosy Department, Faculty of Pharmacy, October 6 University, Egypt. The plant materials (2 kg, moisture content ≤ 10%) were powdered and divided into two portions (1 kg each). Each portion was extracted separately using methanol (80%, 1.5 L, 2 h) in a Soxhlet apparatus under continuous solvent recycling and recovery conditions. After filtration, the solvent was evaporated under reduced pressure (G1-Heidolph, Germany). The resulting extract was defatted using petroleum ether (5 × 500 mL) for removal of waxy and lipoidal maters. After removal of petroleum ether, the *A. azurea* methanol extract (AAME, 210 g, 10.5% w/w) was used in chemical and biological investigations^18^.

### Experimental design for evaluating the biological activities of AAME and reporting its phytoconstituents

The antimicrobial moreover in vitro cytotoxic potential of AAME were assessed. Sulphorhodamine-B (SRB) assay was used to detect cytotoxicity. Cell cycle analysis was used to determine the induction of cell death, whereas acridine orange **(**AO) was used to detect autophagic cell death. The protein expression level of TNFα, Casp3, Casp8, p-JNK, t-JNK, IL-17, p-NFκB, t-NFκB, TRAF6, p-MAPK, t-MAPK, and Ap1 was measured to determine the affected cytotoxicity pathways. Furthermore, the identification of AAME phytoconstituents was conducted using HRLC/T-TOF-MS/MS. Moreover, network pharmacology was done to distinguish the interaction between the AAME metabolites and the IL-17 and TNFα pathways components.

###  Evaluation of AAME cytotoxicity and in combination with Cisplatin

Normal cell line and bone, skin, and oral cancer cell lines **(**HSF, MG63, A375, HNO97 respectively**)** were sourced from the Theodor Bilharz Research Institute, Cairo, Egypt, and were plated in 96-well microplates with RPMI-1640 medium containing 10% FBS and antibiotics. Following a 24-hr incubation period, the cells were exposed to the AAME (0-500 *µ*g/mL) or Cis (0–50 *µ*g/mL) for 48 h^21–23^. “A 48-hour exposure period was selected to allow sufficient time for the induction of cytotoxic and apoptotic responses and to better capture potential synergistic effects between AAME and Cis. Subsequently, the cytotoxicity assessment was conducted on the viable cells using SRB assay^24^. The optical density (OD) of each well was measured at 570 nm spectrophotometrically using an ELISA microplate reader (Sunrise Tecan reader, Germany). Subsequently, IC50 values were calculated. Surviving percentage is the fraction of viable cells compared to the control, where the surviving fraction = OD of treated cells/ OD of control cells*100. Untreated cells served as the control. The synergistic effect of AAME and Cis were assessed through Compusyn software^25^. The combination index (CI) was computed for various treatment combinations in both MG-63 and HNO97 cell lines. The CI values indicated a more favorable synergy in oral cell lines, prompting a focus on the oral cancer model. By utilizing the CI, the nature of the drug interaction between AAME and Cis was established: a CI < 1 implied a synergistic effect, CI = 1 signified an additive effect, and CI > 1 indicated an antagonistic effect. The dose reduction index (DRI) was employed to determine the extent to which the dose of Cis could be decreased in combination therapy compared to monotherapy: a DRI < 1 denoted a negative dose reduction, DRI = 1 indicated no dose reduction, and DRI > 1 indicated a beneficial dose reduction. The dose of AAME and Cis with the lowest CI and highest Cis DRI in HNO97 cells was chosen for further analysis.

### Autophagy detection by flow cytometer

Autophagic cell death is quantitatively evaluated by utilizing AO lysosomal stain combined with flow cytometric analysis. Following treatment with AAME and Cis (500 and 7.045 *µ*g/mL respectively) for 48 h, cells were harvested by trypsinization and rinsed twice with ice-cold Phosphate Buffered Saline (PBS) at pH 7.4. The cells were then stained with AO (10 *µ*M) and kept in the dark (37 °C, 30 min). Subsequently, the stained cells were processed through an ACEA Novocyte™ flow cytometer (ACEA Biosciences Inc., San Diego, CA, USA), and the AO fluorescent signals were examined using the FL1 signal detector (λex/em 488/530 nm). In each sample, 12,000 events were recorded, and the net fluorescent intensities (NFI) were quantified utilizing ACEA NovoExpress™ software.

### Cell cycle determination by flow cytometer

Cells were gathered by trypsinization and fixed in ethanol (4 °C, 70%) overnight post-treatment. Cells were centrifuged (1,000–1,200 rpm, 5 min) and washed twice with cold PBS (pH 7.4) prior to fixation. Following fixation, the cells were suspended in PBS with propidium iodide (PI) (50 *µ*g/mL) and RNaseA (0.1 mg/mL) (Beyotime, Jiangsu, China) after being washed twice with PBS. For DNA staining, PI and RNase A were used to ensure specific measurement of DNA content by eliminating RNA-associated fluorescence interference. The cells were incubated (30 min at 37 °C) in darkness and subsequently evaluated using an ACEA Novocyte™ flow cytometer to determine the distribution of cells in the G1, S, and G2/M phases of the cell cycle.

###  Protein expression level of IL-17 and TNFα signaling pathways components

Ice-cold lysis buffer was utilized to extract the necessary cell lysate. To isolate the proteins from the cell lysate, the proteins underwent electrophoresis on 12% SDS–polyacrylamide gels and were subsequently transferred electrophoretically onto Amersham Hybond P Western blotting membranes (GE10600021 Sigma, Sigma-Aldrich, MO, USA). Following blockage with 5% skim milk, the membranes were probed with primary antibodies, including mouse anti-β-actin, TNFα, Casp3, Casp8, p-JNK, t-JNK, IL-17, p-NFκB, t-NFκB, TRAF6, p-MAPK, t-MAPK, and Ap1 monoclonal antibodies (Santa Cruz Biotechnology, Santa Cruz, California, USA) for 1 h, followed by HRP-conjugated rabbit anti-mouse IgG or HRP-conjugated goat anti-rabbit IgG. The quantification of the protein-bound bands was carried out through image analysis using a ChemiDoc MP imaging system (v3, Hercules, California, USA). Band intensities were quantified using Image Lab software (Bio-Rad), and the expression levels of target proteins were normalized to β-actin as an internal loading control. For phosphorylated proteins, the ratios of phosphorylated to total protein (e.g., p-JNK/t-JNK, p-NFκB/t-NFκB, and p-MAPK/t-MAPK) were calculated. All measurements were obtained from at least three independent experiments, and densitometric values were expressed as relative fold change compared to the control group. Statistical analysis of band intensities was performed as described in the statistical analysis section.

### Network pharmacology study on A. auzrea main metabolites

####  identification of *AAME* constituents and detection of the main targets

Rosmarinic acid, quercetin, and linolenic acid were selected for the network pharmacology analysis, based on their prominence both in the literature and in our experimental findings. These metabolites are well-documented as major bioactive constituents of *A. azurea*, with established pharmacological relevance, particularly in anti-inflammatory and anticancer pathways. In addition, our metabolomic analysis indicated that these compounds were among the most abundant and/or biologically significant constituents in the AAME. Rosmarinic acid, quercetin, and linolenic acid were entered into the PubChem database to retrieve their SMILES notations (https://pubchem.ncbi.nlm.nih.gov/, accessed on 12 Nov. 2024)^26^. The chemical structures were then downloaded for further analysis. The SMILES strings of the metabolites were input into Swiss Target Prediction (http://www.swisstargetprediction.ch/, accessed on 14 Nov. 2024)^27^ to identify the potential genes associated with each compound. After obtaining the target genes, any duplicate genes were removed to generate a refined list of unique target genes.

#### Identification of cancer-related genes

The genes relevant to oral cancer were obtained from the GeneCards database (https://www.genecards.org/,accessed on 15 Jan. 2025)^28^. We searched and gathered genes in the GeneCards database using the phrase “oral cancer” and chose genes with a relevance score of 5 indicating that the gene is highly relevant to “oral cancer”, reflecting a strong connection between the gene and these cancer types, and suggesting that the gene plays an important role in their biology or potential treatment.

####  Venn diagram analysis

A Venn diagram was constructed using the Jvenn plug-in cancer (https://bioinfogp.cnb.csic.es/tools/venny/, accessed on 16 Jan. 2025) to visualize the overlap between the genes associated with the active constituents of AAME and oral cancer. The diagram revealed the common genes shared between the AAME metabolites and oral cancer.

#### Network construction and visualization

Using Cytoscape V3.10.2^29^., a network visualization was created to map the relationships between AAME metabolites, their associated genes, and the common cancer-related genes. *A. azurea* and its metabolites were placed at the center, with the genes targeted by these metabolites surrounding them. The targets were colored according to their probability, indicating the strength of the potential interaction between a metabolite and a target protein. The shared genes between the *A. azurea* metabolites and the cancer-related genes were drawn as inverted triangles.

####  Enrichment Analysis: Gene Ontology (GO) and kyoto encyclopedia of genes and genomes (KEGG) pathways

To explore the biological significance of the common genes, GO and KEGG pathway enrichment analyses were performed. ShinyGO http://bioinformatics.sdstate.edu/go/, accessed on Feb. 2025)^30^ was used to generate a lollipop chart for the GO analysis, revealing biological processes and pathways associated with the common genes.

#### Protein-protein interaction

The STRING protein-protein interaction database was used to examine the interconnectivity within the chosen target genes^31^.

### Antibacterial activity

The antibacterial activity of the AAME was evaluated against two pathogenic bacterial strains: *Staphylococcus aureus* (ATCC 25923) and *Acinetobacter baumannii* (AB5075). These microbial strains were provided by the Molecular Biology Department, National Research Centre and Microbiology Laboratory, Faculty of Pharmacy, Cairo University, respectively. The bacteria were cultured on Mueller-Hinton agar and incubated (20 h, 37 °C).

###  Agar disk diffusion assay and minimum inhibitory concentration (MIC)

The antimicrobial test was performed using the agar disk diffusion method^32^, with minor modifications. Mueller-Hinton agar plates were inoculated by swabbing with standardized bacterial suspensions (2 × 10^8^ CFU/mL). AAME (50 mg/mL in dimethyl sulfoxide (DMSO)) was sterilized by syringe filter (0.22 *µ*m) (Sigma-Aldrich, USA). Sterile paper discs (6 mm diameter) were loaded with AAME (20 *µ*L). The discs were then placed on the surface of the inoculated agar plates. Ciprofloxacin (10 mg/disc) served as positive control, while discs containing only DMSO were used as the negative control. The plates were incubated at 37 °C for 24 h, after which the diameters of the inhibition zones were measured to assess antibacterial activity.

The MIC, the lowest concentration of AAME that showed no visible bacterial growth, was determined using the broth dilution method^33,34^. In brief, a stock solution of AAME was prepared (100 mg/mL DMSO). Two-fold serial dilutions in double-strength Mueller–Hinton broth (Sigma Aldrich, United States) were done. To each dilution tube 100 *µ*L was added of the bacterial suspension (~ 10⁶ CFU/mL), prepared from a 24-h culture grown on Tryptic Soy Agar (TSA) at 37 °C. Positive and negative controls were included.

### Determination of phenolic and flavonoids contents

The total phenolic content (TPC) using a colorimetric assay based on the Folin-Ciocalteu method^35^. AAME (0.5 mL, 1 mg/mL in distilled water) was reacted with Folin-Ciocalteu reagent (0.25 mL), distilled water (5 mL), and sodium carbonate solution (7%). The sample was allowed to stand at 25 °C for 30 min in the dark. A blank was prepared using distilled water instead of the AAME for control. The absorbance was determined by measuring with a spectrophotometer, DlaB (Model sp-v1000) at 730 nm in triplicate, and the mean absorbance was obtained. The TPC was reported as milligrams of gallic acid equivalents per gram of the extract (mgGAE/gE) using the calibration curve of the standard gallic acid (1–50 mg/mL).

The total flavonoid content (TFC) was measured using the aluminum chloride colorimetric assay, as described by Bahorum et al., with certain adjustments^36^. AAME (300 mg) was diluted with acetone (99.7%, 20 mL) and hydrochloric acid (30–33%, 2 mL) in a volumetric flask. The mixture was boiled under reflux (100 °C, 30 min), filtered, and allowed to cool. Acetone was added till the final volume equal 100 mL. The AAME stock solution (3 mg/mL, 0.5 mL) was mixed with distilled water (2.5 mL), followed by sodium nitrate (5%, 0.5 mL). After 5 min, aluminum chloride (10%) was added, and the solution was allowed to stand for another 5 min. The absorbance was determined at 510 nm using a spectrophotometer (DlaB, Model sp-v1000), in triplicate versus the blank (using distilled water instead of aluminum chloride). The TFC was reported as milligrams of rutin equivalents per 100 g of extract (mgRE/100gE) using the calibration curve of the standard rutin (1–50 mg/mL).

HPLC analysis was carried out using an Agilent 1260 series. The separation was carried out using Zorbax Eclipse Plus C8 column (4.6 mm x 250 mm i.d., 5 *µ*m). The mobile phase consisted of water (A) and 0.05% trifluoroacetic acid in acetonitrile (B) at a flow rate 0.9 mL/min. The injection volume was 5 *µ*L. The column temperature was maintained at 40 °C. The mobile phase was programmed consecutively in a linear gradient as follows: 0 min (82% A); 0–1 min (82% A); 1–11 min (75% A); 11–18 min (60% A); 18–22 min (82% A); 22–24 min (82% A). The multi-wavelength detector was monitored at 280 nm in triplicate, and the mean ± SE was recorded. The method was carefully validated according to guidelines provided by International Conference on Harmonization (ICH) of Technical Requirements for Pharmaceuticals for Human Use^37^.

###  HRLC/T-TOF- MS/MS analysis of AAME

The analysis was proceeded at the Proteomics and Metabolomics department of the Children’s Cancer hospital − 57,357 in Cairo, Egypt. The analysis utilized an Exion LC triple TOF 5600 + system (SCIEX, Framingham, MA, USA) operated at 40 °C. The system was equipped with a precolumn (Phenomenex, In-line filter disks, 0.5 *µ*m x 3.0 mm) and an X select HSS T3 C-18 column (Waters Corporation, Milford, CT, USA, 2.5 *µ*m, 2.1 × 150 mm). The AAME (50 mg) was dissolved in a solvent working solution (Water: Methanol: Acetonitrile-50:25:25, 1000 *µ*L), kept on a vortex for 2 min followed by its ultra-sonication for 10 min. It was then centrifugated (10 min at 10000 g). The phytochemical constituents of the AAME were analyzed using HRLC/T-TOF-MS/MS in negative ionization mode. Samples (2.5 *µ*g/*µ*L, 10 *µ*L) were injected using the following mobile phases (MP): MP; A was ammonium formate buffer (5 mM, pH 8) containing methanol (1%), while MP; B was acetonitrile (100%). The gradient elution was performed according to Table 1. The working solvent was injected as a blank sample. The constituents detected were recorded by Analyst TF 1.7.1 software (Sciex Framingham, MA, USA), Peak View software (Sciex Framingham, MA, USA), and MS-DIAL 4.6 software (RIKEN, Japan) for data analysis. Metabolites were identified based on their R_t_, *m*/*z*, data, MS^2^ spectra and searching the phytochemical dictionary of natural database (CRC, Wiley).

**Table 1:** Mobile phase composition for gradient elution in HRLC/T-TOF-MS/MS analysis of AAME.

Time (min)	Flow rate (mL/min)	Mobile phase A %	Mobile phase B %
0	0.3	95	5
1	0.3	95	5
21	0.3	5	95
28	0.3	5	95
28.1	0.3	95	5
35	0.3	95	5

### DPPH radical scavenging assay

Rutin and gallic acid were used as standards. Stock solutions (2 mg/mL in methanol) were prepared followed by serial dilutions (5-100 *µ*g/mL). Each dilution (0.1 mL) was mixed with a freshly prepared DPPH (2 mL) and the absorbance was measured using a colorimeter at 515 nm after a 30 s reaction. Positive control and a blank were used in the experiment^8^. The percentage antioxidant activity (%AA) was primarily deduced from the following equation: 100 – [(Ab sample – Ab negative control) x 100 / Ab positive control]. The experiments were carried out in triplicate and the results were expressed as mean ± standard error (SE). All measurements were done in triplicates.

### Statistical analyses

Statistical analyses were conducted using GraphPad Software V 9.0 (San Diego, CA, USA). Normality was assessed using the Kolmogorov–Smirnov test. To compare between two or more groups of normally distributed data, t-test or One-way ANOVA followed by tukey or games howel as post hoc tests were used, respectively.

## Results and discussion

###  Cytotoxic impact of *A. azurea* and its synergy with cisplatin

**Fig. 1:**
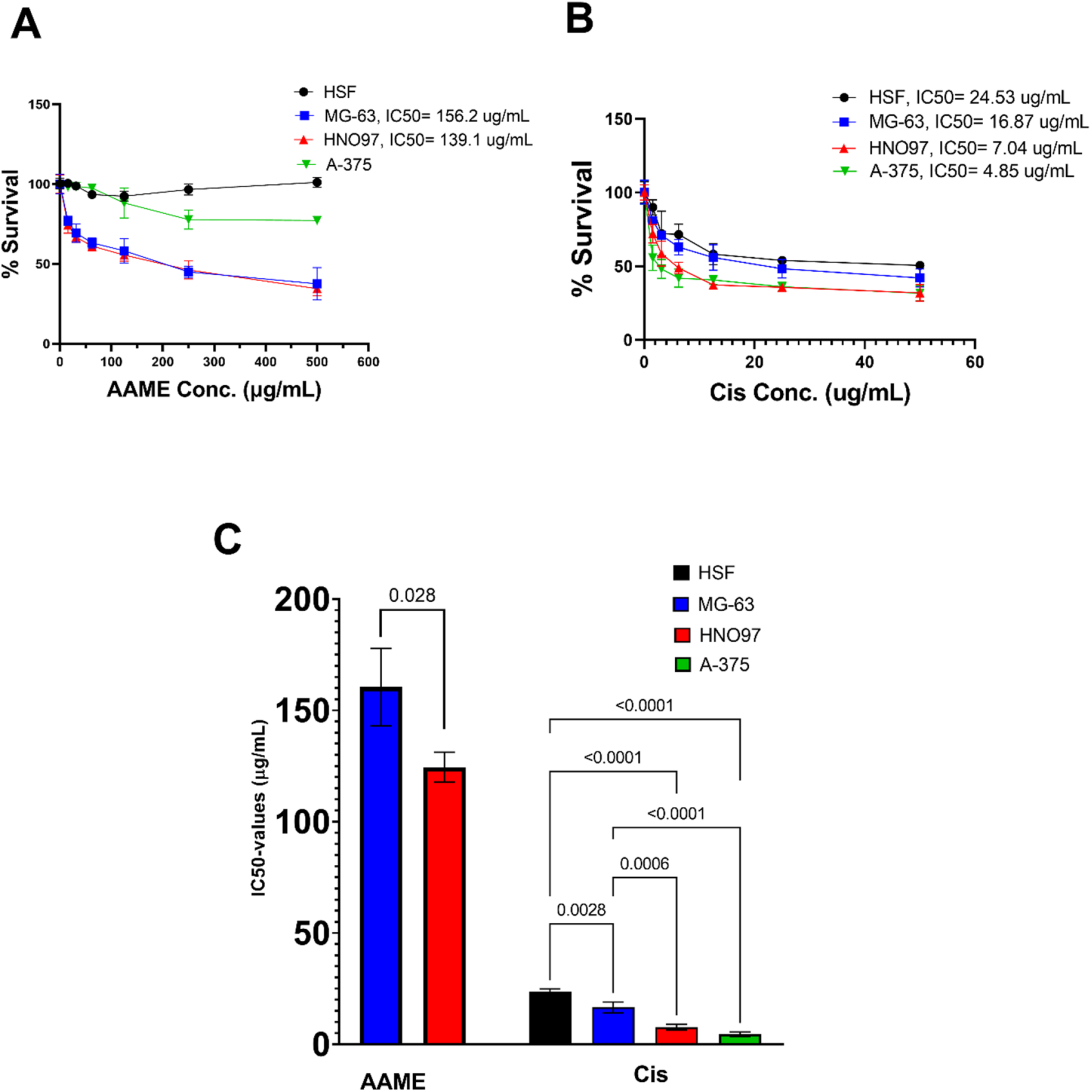
Effect of AAME (**A**) and Cis (**B**) on the viability of cancer cells (A-375, HNO97, and MG-63) and normal cells (HSF). Comparison of IC50 values between the various cell lines treated with AAME or Cis (**C**). The presented values represent the mean ± standard deviation (SD) from two independent experiments conducted in triplicate. The surviving % denotes the percentage of viable cells relative to the control, calculated as the optical density of treated cells divided by the optical density of untreated cells. IC50 values were calculated from dose inhibition curves of nonlinear regression.

The SRB assay provides valuable insights on the cytotoxic effects of AAME and Cis on normal cell line compared to bone, oral, and skin cancer cell lines. AAME shows dose dependent cytotoxicity on both MG-63 bone and HN097 oral cells with IC50 values of 156.2 *µ*g/mL and 139.2 *µ*g/mL, respectively, however it has no cytotoxicity against A-375 and a high safety profile on HSF normal cell lines (Fig. 1A). Similarly, AAME exhibited no cytotoxicity against several cell lines, including MCF-7, HepG2, WEHI, and MDBK^38^. Figure 1B presents the dose dependent cytotoxic effect of Cis showing IC50 values equal 24.53, 16.87, 7.045, and 4.85 *µ*g/mL on HSF, MG-63, HNO97, and A-375, respectively. Comparing the IC50s in Fig. 1C, AAME showed higher cytotoxicity in HNO97 compared to MG-63 cells (*P* < 0.05), while Cis was more potent in A-375 cells with IC50 4.85 *µ*g/mL. The IC50 values are summarized in Table S1.

**Fig. 2:**
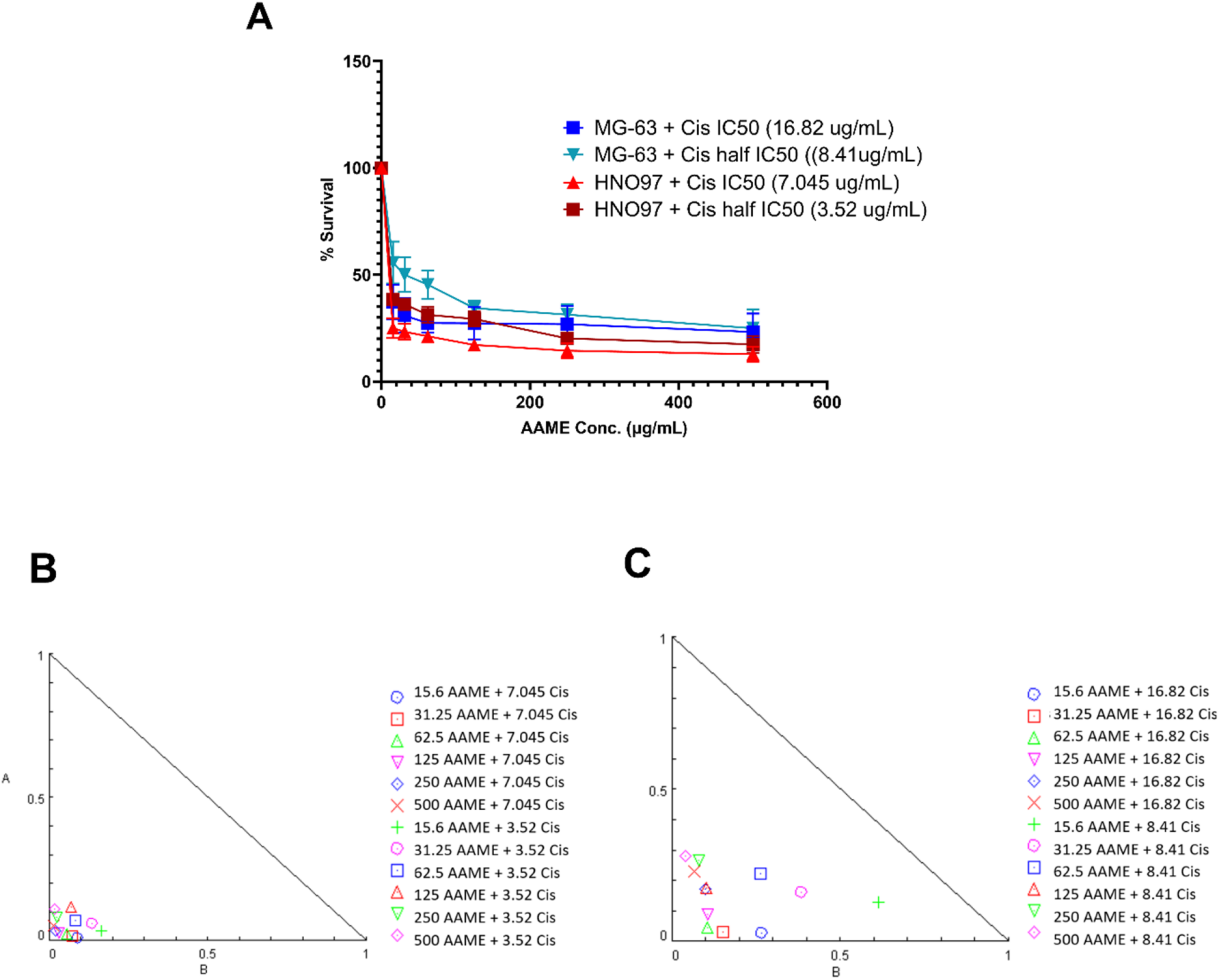
The surviving fractions of HNO97 and MG-63 cells treated with IC50 and half IC50 values of Cis in combination with different concentrations (0-500 *µ*g/mL) of AAME for 48 h (**A**). A normalized isobologram generated by Compusyn for HNO97 (**B**) and MG-63 (**C**) cell lines. Data used in Compusyn was the mean of two separate experiments performed in triplicate.

Several natural metabolites exhibited anticancer effect against oral and bone cancer cells^39,40^. The benefits of combining plant extracts with Cis have shown promise in alleviating Cis toxicity, improving efficacy, and reducing drug resistance^41^. Due to the effectiveness have AAME on HNO97 and MG-63 cells, further combination analysis were done on both cell lines. Figure 2A showed the effect of using IC50 and half IC50 of Cis in combination with different concentrations of AAME on HNO97 and MG63 cell lines. The combination showed a higher inhibition effect when AAME was combined with Cis compared to monotherapy; potentially enhancing overall treatment outcomes. Combining AAME (500 *µ*g/mL) with the IC50 dose of Cis led to an 80% decrease in the viability of HNO97 cells compared to using Cis alone. Similarly, the co-administration of AAME (15.625 *µ*g/mL) with half the IC50 concentration of Cis led to a cell viability inhibition of 70%, indicating a decrease in the Cis dose by approximately 71.84%. The CI and DRI for different doses were calculated to evaluate the synergy between AAME and cis (Fig. 2B&C, Table S2). In HNO97 cells, the combination of AAME at a concentration of 250 *µ*g/mL with 7.045 *µ*g/mL of Cis resulted in a cell viability inhibition of 86%. This combination exhibited the lowest CI value of 0.055 and a notably high DRI for Cis, suggesting it as the most efficacious combination with a strong synergistic effect. Therefore, this combination could potentially help reduce the required dose of cisplatin, thereby minimizing its associated side effects and was further used in the following biological tests.

###  The effect of AAME or/and Cis on the protein expression levels of IL-17 and TNFα signaling pathway

**Fig. 3:**
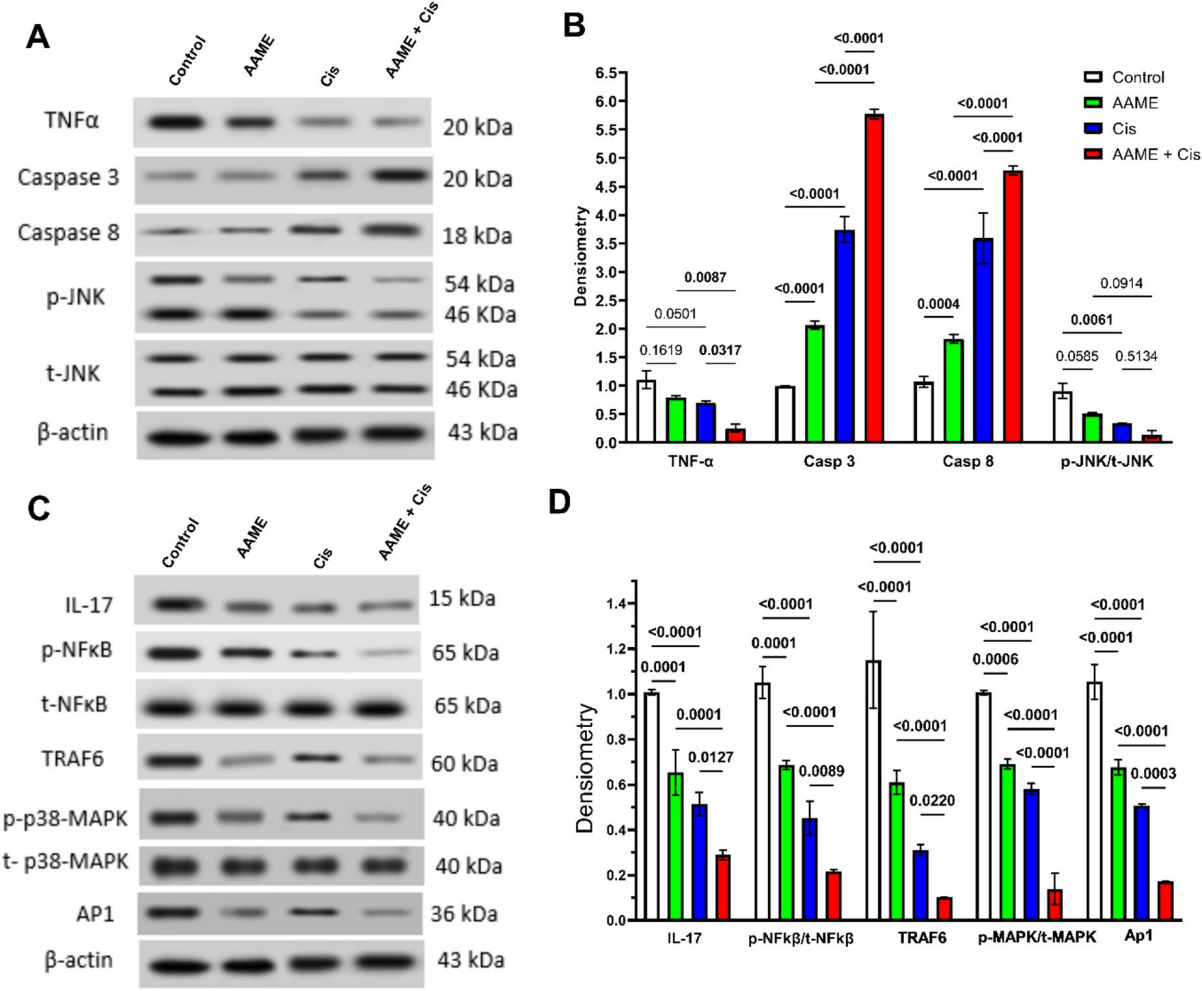
The Protein expression levels of TNF-α and IL-17 signaling pathway components using Western blot analysis. Western blot images of TNFα, Casp3, casp8, t/p-JNK (**A**) and IL-17, t/p-NFκB, TRAF, t/p-MAPK, and AP1 (**C**). β-actin was used as control. The relative expression levels of proteins measured using densitometric analysis (**B**&**D**). The presented values represent the mean ± SD from two independent experiments conducted in triplicate.

Elevated TNFα levels in the tumor microenvironment of oral squamous cell carcinoma have been shown to promote tumor invasion by enhancing both the pro-inflammatory and pro-invasive characteristics of these cells. Additionally, TNFα facilitates a paracrine signaling mechanism that recruits and activates inflammatory cells. Genetic polymorphisms that influence TNFα expression are strongly linked to an increased susceptibility to oral cancer^42,43^. HNO97 cells treated with AAME or Cis showed a significant decrease in TNFα,, p/t-JNK ratio and an increase in Casp3, Casp8 protein expression levels, furthermore their combination resulted in a more pronounced modulation of these protein levels (Fig. 3A&B). These findings suggest a potential involvement of TNFα-related signaling modulation rather than direct functional inhibition. Flavonoids have been identified as potential TNFα inhibitors^44^. Rosmarinic acid, quercetin, and linolenic acid, the detected compounds in *A. azurea*, have been reported to be associated with sensitization of cell death, potentially through modulation of TNFα signaling^45–47^.

The IL-17 signaling pathway exhibits an essential in cancer, contributing to tumor promotion through driving inflammation and modulating the tumor microenvironment thereby affecting cellular survival, growth, and the potential for metastasis. Moreover, the IL-17 pathway presents opportunities for targeted cancer immunotherapy^48^. IL-17 is highly expressed in oral cancer and plays a vital role in the pathogenesis of oral squamous cell carcinoma^49^. Treating cells with either AAME (250 *µ*g/mL) or Cis (7.045 *µ*g/mL) lead to a significant decrease in the protein expression level of IL-17, pNFκB/tNFκB ratio, TRAF6, pMAPK/tMAPK ratio, and AP1. Combining AAME and Cis further enhanced these observed changes at the protein level compared to each one alone (Fig. 3C&D). These results indicate a possible modulation of the IL-17 signaling pathway; however, this remains correlative and requires further functional validation. Natural products have the potential to modulate IL-17 signaling, influencing its dual roles in mediating protective immune responses and contributing to pathogenic inflammation^50^. Future studies employing functional approaches, such as pathway inhibition, gene silencing, or overexpression strategies, are necessary to validate the mechanistic role of these pathways and confirm their direct involvement in the observed anticancer effects.

### Cell cycle analysis

**Fig. 4:**
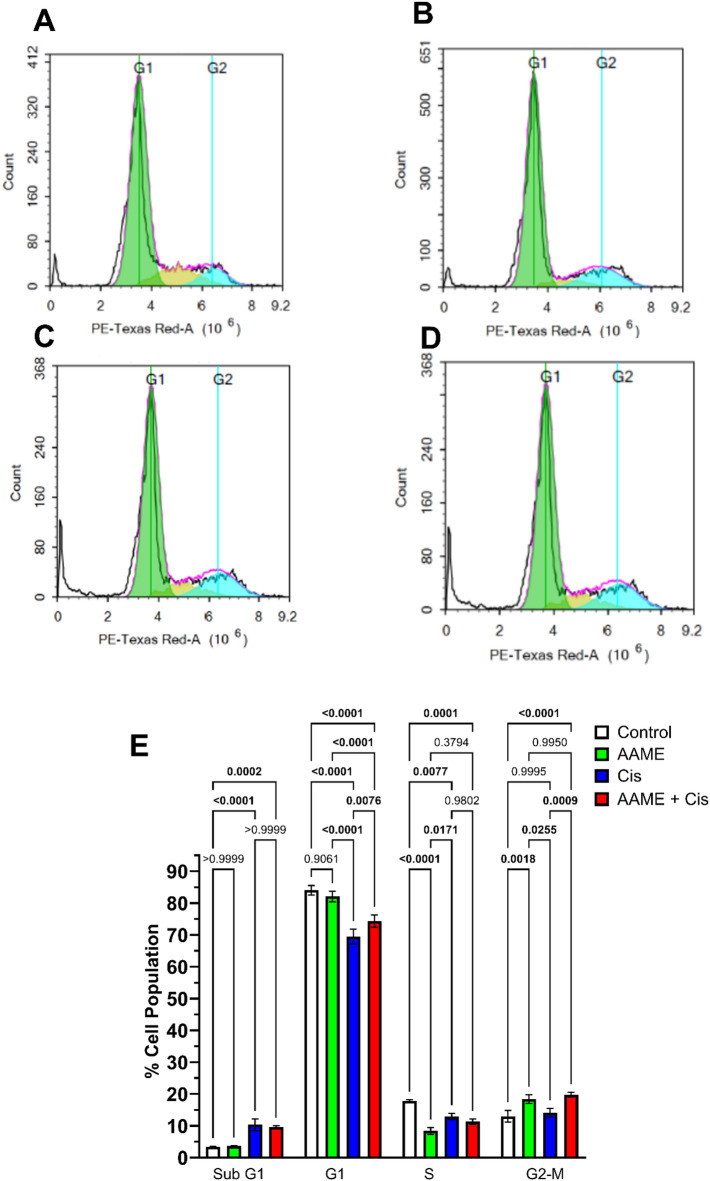
Cell cycle analysis by flow cytometry for HNO97 treated with AAME and/ or Cis for 48 h: (**A**) control, (**B**) AAME, (**C**) Cis. (**D**) AAME + Cis. (**E**) Comparative analysis for the sub-G1, G1, S, and G2-M phases across different groups. All results are expressed as percentages of the cell population with mean ± SD of three experiments.

Plant extracts exhibit cytotoxicity through various mechanisms, including cell cycle interruption. The impact of AAME on the cell cycle distribution of HNO97 cells was studied to determine if the antiproliferative effect induced was associated with cell cycle arrest. Treatment with AAME or Cis altered the distribution of cell cycle phases significantly (Fig. 4). A higher proportion of cells treated with AAME were in the G2/M phase compared to the control group (12.97% vs. 18.42%). Cis treatment led to cell cycle arrest at the Sub G1 phase in contrast to the control group (10.34% vs. 3.43%). The combination of AAME and Cis induced cell cycle arrest at two phases: Sub G1 and G2/M (Sub G1: 9.68% vs. 3.43%, G2/M phase: 19.79% vs. 3.43%). Evaluating the potential of anticancer agents to prompt cell cycle arrest in cancer cells is a common practice. For instance, rosmarinic acid regulates the cell cycle by arresting the G0/G1 phase in MDA-MB-231 cells and the S-phase in MDA-MB-468 cells^51^. Additionally, quercetin inhibits cell proliferation by inducing G1 cell cycle arrest^52^.

###  Autophagy analysis

**Fig. 5:**
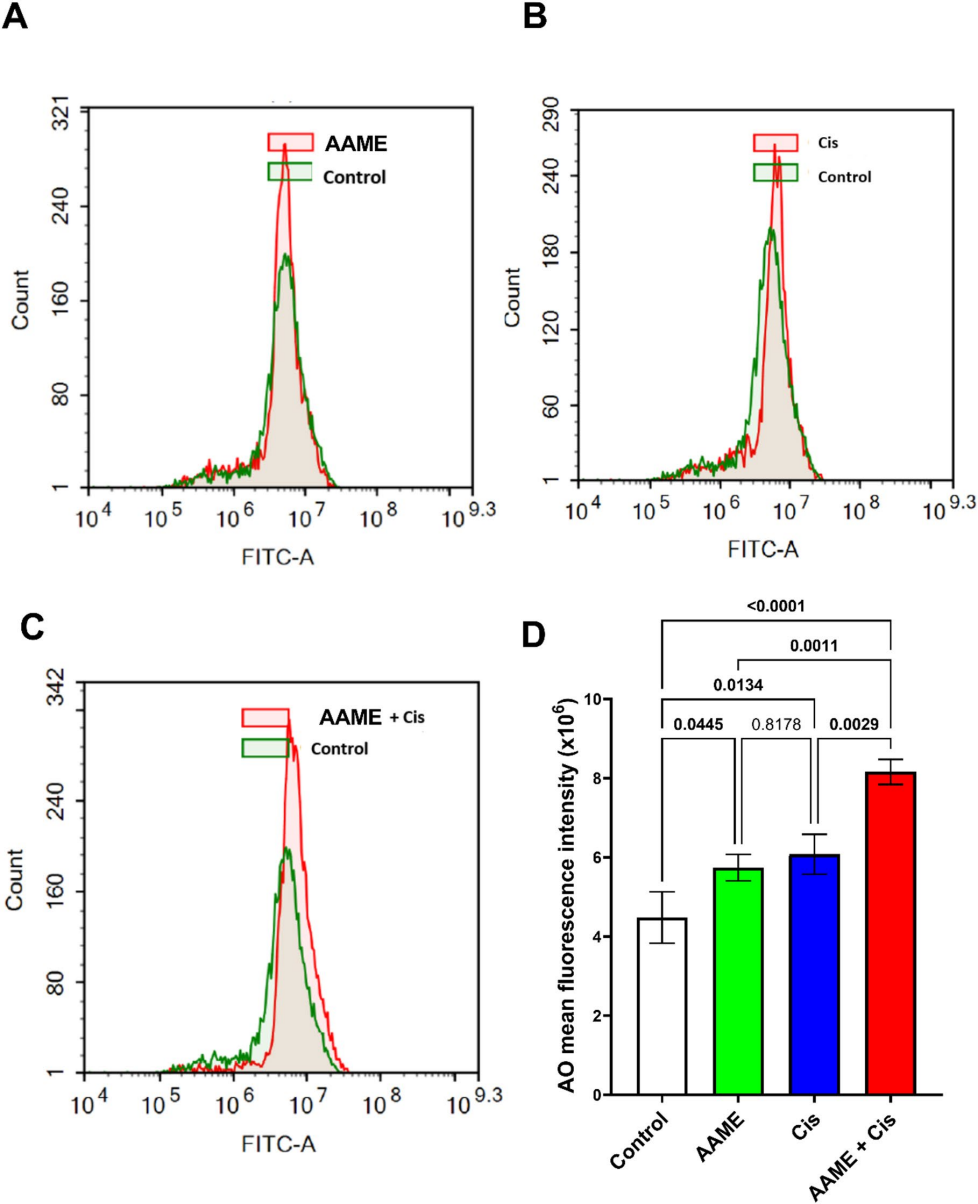
Autophagy analysis by flow cytometry for HNO97 treated with AAME and/ or Cis for 48 h: (**A**) AAME, (**B**) Cis., (**C**) AAME + Cis. (**D**) Comparative analysis for mean ± SD of fluorescence intensity across different groups.

Autophagy is the basic cellular machinery involving the digestion of damaged cellular components. Excessive and sustained autophagy can result in tumor cells’ death and shrinkage. Literature has demonstrated that numerous anticancer phytoconstituents have the capacity to trigger autophagy in cancerous cells^53^. AAME and Cis induced autophagy in HNO97 cells compared to the control group at P-value equals 0.04 and 0.013, respectively. The combination of AAME and Cis showed further increase in the autophagy as expressed by the AO mean fluorescent intensity compared to control group, AAME, and Cis treated cells (Fig. 5). It was previously reported that Cis can trigger the autophagic response in A549 cells in a time-dependent manner^54^. Although autophagy induction was correlated with Cis resistance in oral squamous cell carcinoma, kaempferide, a flavonoid detected in AAME, mitigates Cis-induced kidney toxicity by suppressing oxidative stress and promoting autophagy^53,55,56^.

###  Network pharmacology

Network pharmacology is performed to explore the potential interaction between AAME main metabolites and the target pathways affecting oral cancer and to identify putative pathways through which the active metabolites exert their effects, offering insights into the complex interactions within biological systems and supporting the generation of potential therapeutic hypotheses.

#### Venny and Cytoscape

**Fig. 6:**
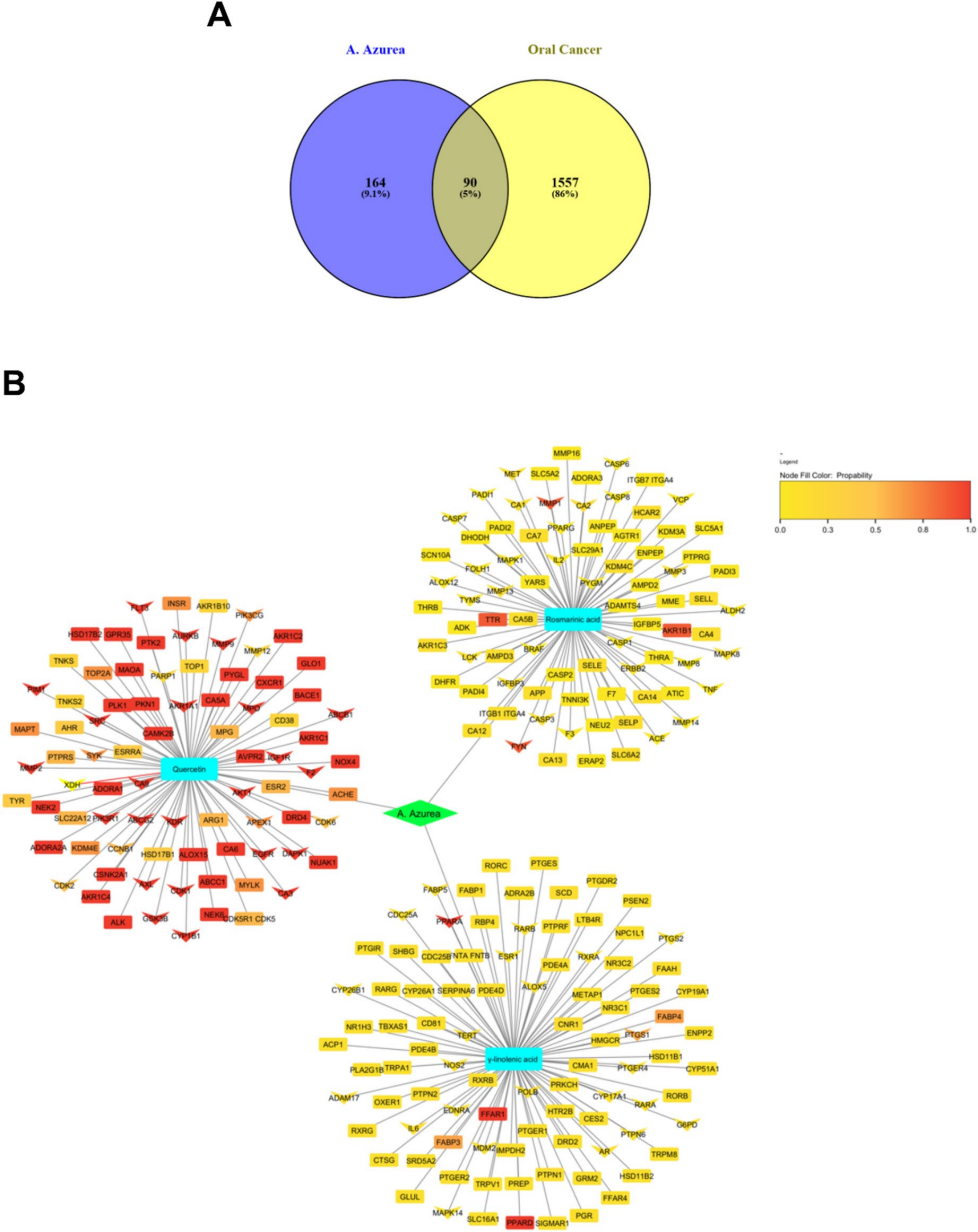
A Venn diagram analysis revealed 90 common genes shared among the datasets: oral (1,647 genes), and AAME (245 genes) (**A**). Cytoscape diagram represents AAME as green diamond, while its active constituent is depicted as blue rounded rectangles (**B**). The target genes are color-coded from yellow to red based on the probability score, with the 90 shared genes between the datasets indicated by inverted triangles, highlighting their potential involvement in mediating the effects of AAME.

In the Venn diagram Fig. 6A, genes associated with oral cancer (1647 genes), and AAME (254 genes) were analyzed, identifying 90 genes that are common. Additionally, in Cytoscape, all the genes related to the active metabolites of AAME, rosmarinic acid, quercetin, and γ-linolenic acid, were added to map their interactions. The Cytoscape network illustrates the predicted relationships of these genes with these metabolites using probability score. The probability score represents the likelihood or confidence level that a certain compound interacts with a particular target. Higher probability scores typically suggest a higher likelihood of a true interaction. Moreover, the 90 genes that are common between AAME active constituents and the oral cancer were identified as potential shared targets (Fig. 6B).

#### SHINY GO and KEGG Pathway Enrichment analyses

**Fig. 7:**
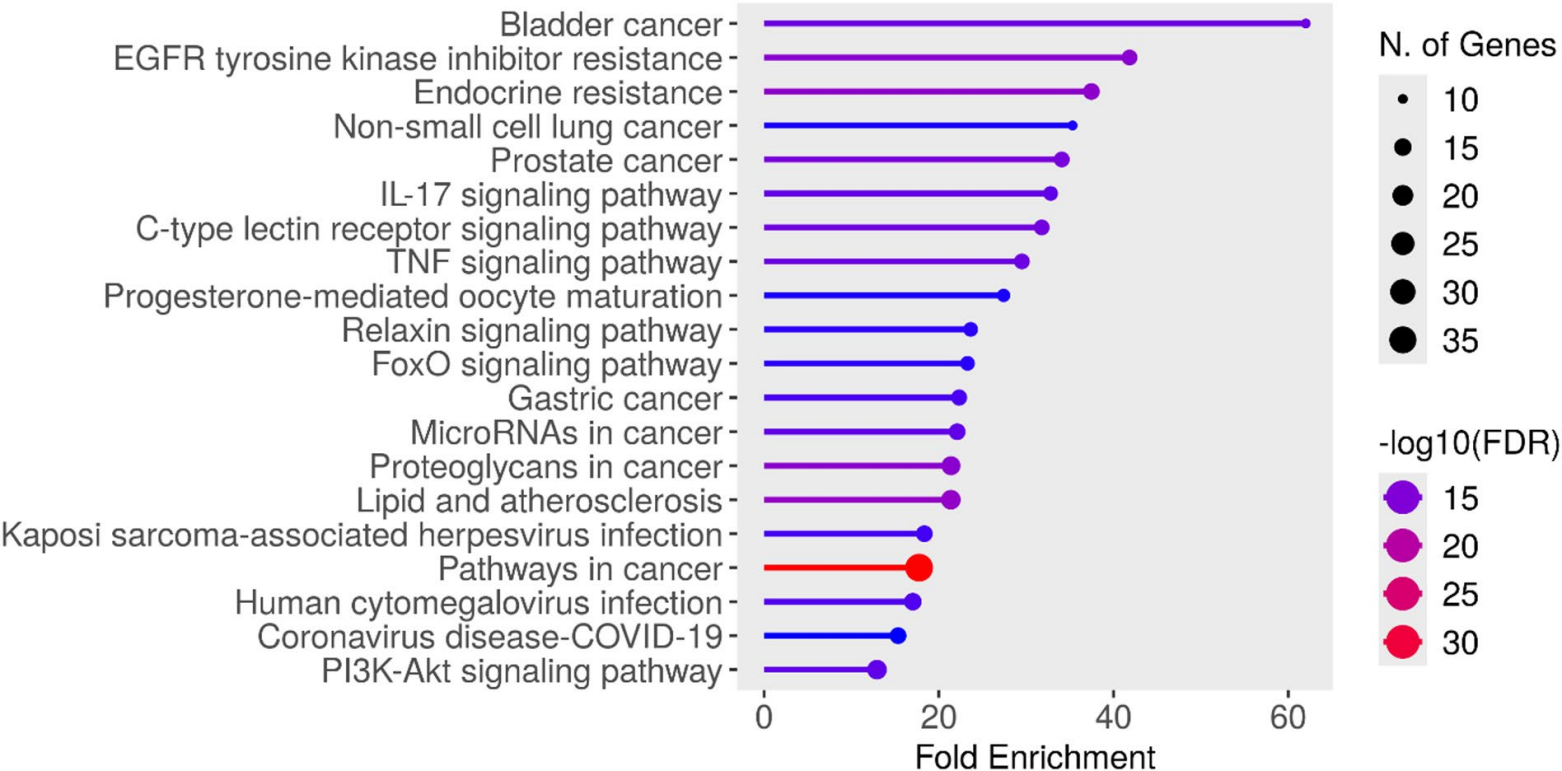
Pathway enrichment analysis of the genes showing the top enriched pathways. Each bar represents a specific pathway, with fold enrichment on the x-axis. Dot sizes indicate the number of genes associated with the pathway, and colors represent the -log10 (FDR), highlighting statistical significance. The x-axis represents fold enrichment, highlighting the degree to which these pathways are overrepresented, while the y-axis lists the pathways ranked by significance.

Figure 7 and Table S3 present a lollipop chart and table illustrating the pathway enrichment analysis results for the shared genes between oral cancer and AAME targets. The top 20 most significant pathway were chosen, among which IL-17, TNFα, Relaxin, FoxO, and Pl3l-Akt signaling pathways were identified as enriched pathways. These pathways are particularly significant in cancer biology. The PI3K-Akt pathway regulates cell growth, survival, and metabolism, and its dysregulation is linked to tumor progression and chemotherapy resistance^57^. The FoxO signaling pathway governs apoptosis and cell cycle advancement in oral squamous cell carcinoma, while also overseeing epithelial-mesenchymal transition, autophagy, stress response, and stem cell characteristics^58^. Similarly, the Relaxin pathway promotes cancer metastasis by influencing the tumor microenvironment, including angiogenesis and extracellular matrix remodeling^59^.

**Fig. 8:**
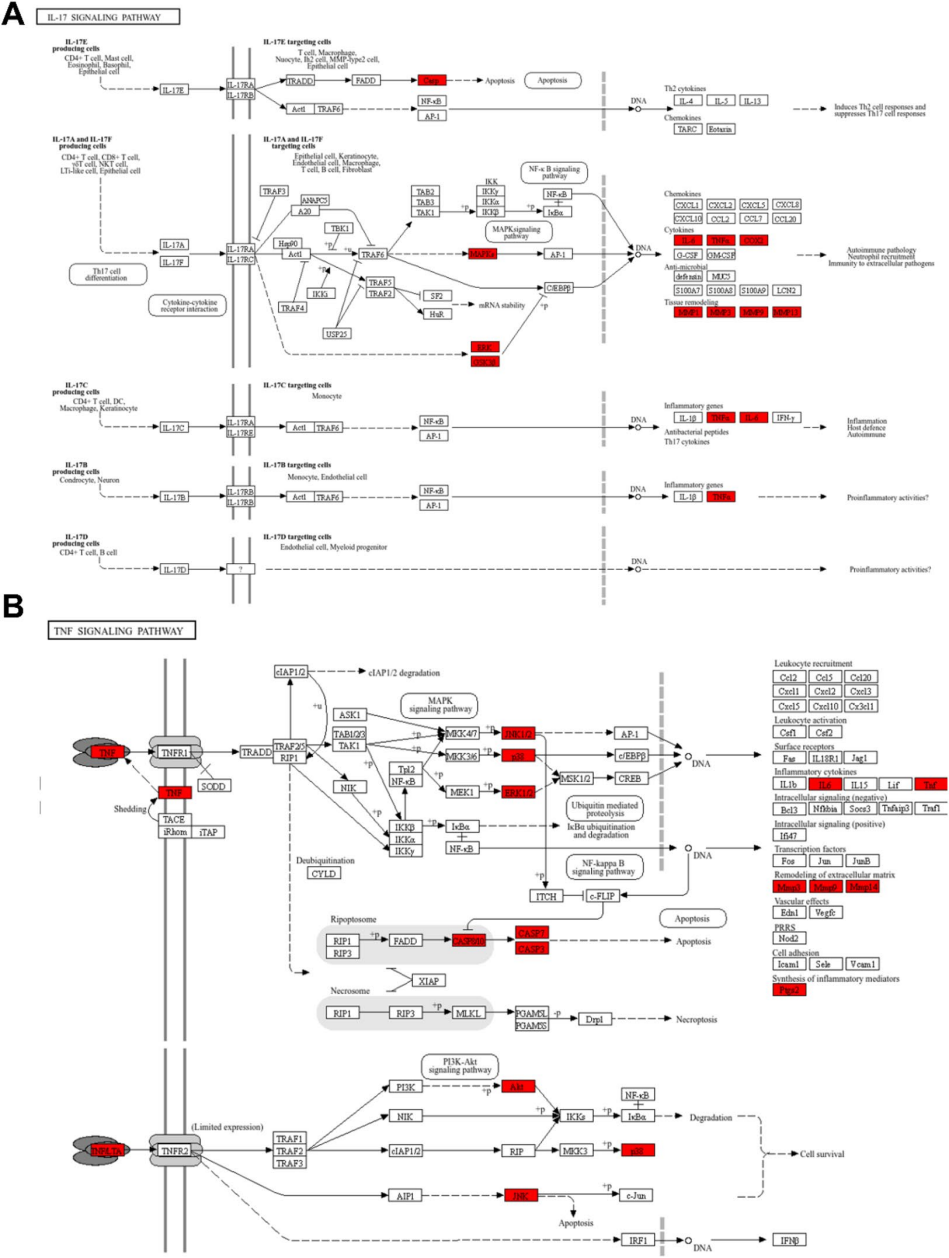
IL-17 signaling pathway diagram (**A**) and the TNFα signaling pathway diagram (**B**). The IL-17 pathway is crucial for immune cell development and survival, with dysregulation linked to cancer progression. On the other hand, the TNFα pathway plays a key role in inflammation and immune response, promoting cell survival and proliferation in tumor environments. The common genes are highlighted in red.

IL-17 and TNFα signaling pathways were among the highly enriched pathways, showing high fold enrichment, significant gene involvement (20 genes, p-value = 1 × 10⁻¹⁵), in addition to their critical roles in cancer biology, promoting inflammation, tumor progression, and metastasis (Fig. 8A&B). TNFα is a target that has been reported to be associated withNF-KB signaling pathway, contributing to cancer progression^60^. Elevated TNFα levels in the tumor microenvironment of oral squamous cell carcinoma have been shown to promote tumor invasion by enhancing both the pro-inflammatory and pro-invasive characteristics of these cells. Kaempferol, that detected in AAME, has been reported as a potential TNF-α modulator^44^. Rosmarinic acid, quercetin, and α-linolenic acid, amonge the identified metabolites in *A. azurea*, have been associated with sensitization of cell death, potentially through modulation of TNFα signaling^45–47^. The IL-17 signaling pathway plays a critical role in cancer by promoting inflammation, modulating the tumor microenvironment, influencing cellular behavior, and offering potential for targeted cancer immunotherapy^48^. Rosmarinic and γ-linolenic acids have been suggested to contribute to anti-inflammatory effects, potentially through modulation of IL-17 signaling^61^. It is important to note that these pathway enrichment results are derived from in silico analyses and therefore represent predictive associations rather than experimentally validated mechanisms.

#### Protein-protein interactions

**Fig. 9:**
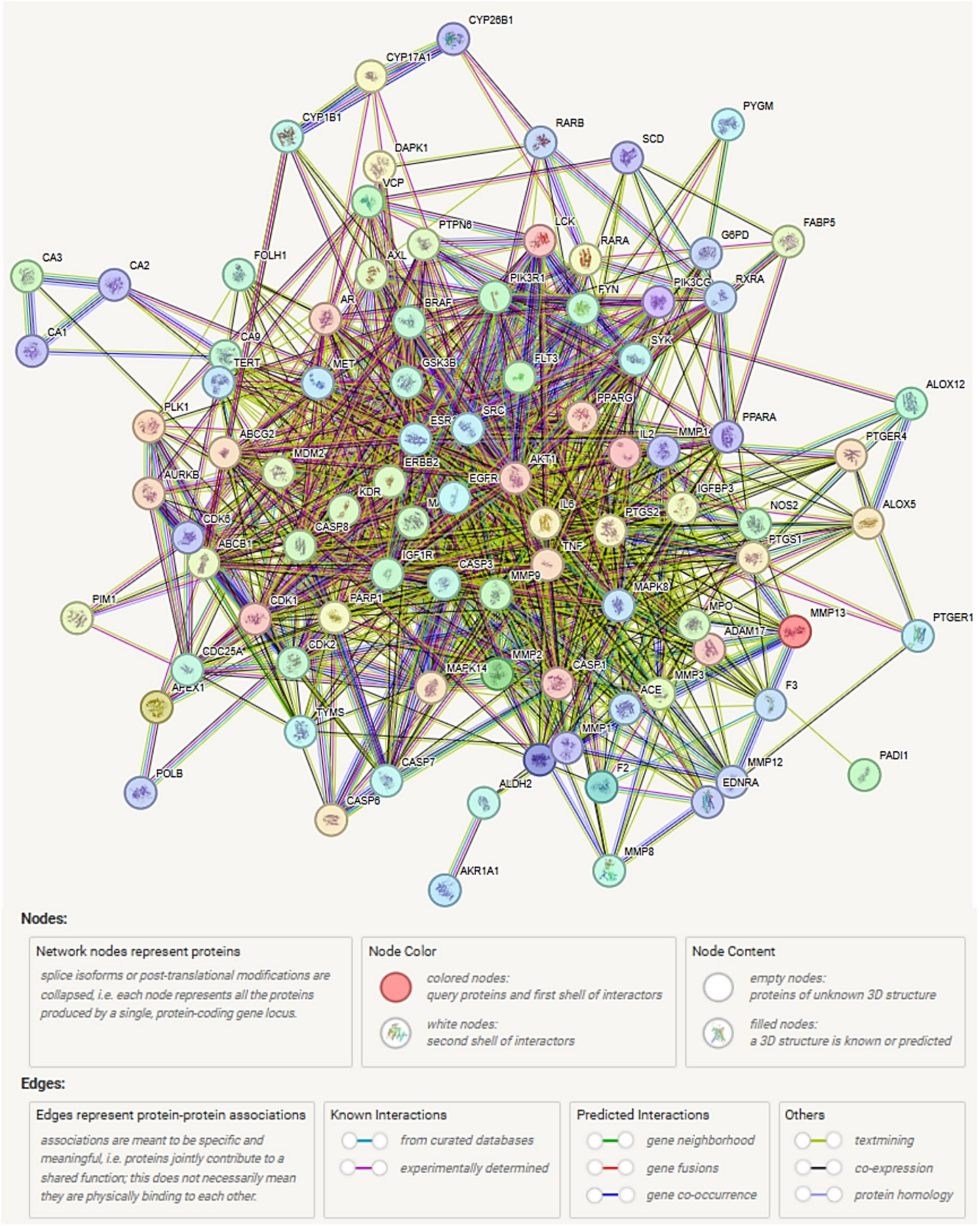
STRING protein-protein interaction analyses. The network has 987 edges (vs. 383 expected edges); enrichment *p*-value < 0.0001; clustering coefficient: 0.646. The thickness and color of the connecting edges denotes the strength and the type of interaction.

Utilizing the STRING protein-protein interaction database^31^, we examined the interconnectivity within the common targets of AAME main ingredients with oral cancer. Employing an interaction score threshold of 0.7 (indicating high confidence), the STRING PPI analysis revealed a densely clustered network (clustering coefficient: 0.646) comprising 90 nodes connected by 987 edges (with an anticipated number of edges being 383). This outcome suggests a significantly heightened level of interaction compared to what would be anticipated for a random set of similar size sourced from the genome (enrichment *p*-value < 0.0001, Fig. 9). However, these interactions are computationally inferred and should be interpreted with caution until validated through experimental approaches.

### Antimicrobial activity

In addition to assessing the anticancer activity, the antimicrobial potential of AAME was evaluated to provide a broader characterization of its biological properties. AAME was tested against two clinically significant bacterial pathogens: *Staphylococcus aureus* and *Acinetobacter baumannii* using the agar disk diffusion and broth dilution method. The *S. aureus* strain used in the study is known to be susceptible to multiple antibiotics, including ciprofloxacin, whereas *A. baumannii* exhibits resistance to many conventional antimicrobials. AAME showed moderate activity against *S. aureus* in comparison with ciprofloxacin (inhibition zone equal to 16 and 34 mm respectively). Complete inhibition of *S. aureus* growth was achieved at 12.5 mg/mL (MIC). In contrast, AAME didn’t show any inhibitory effect against *A. baumannii* (Suppl. Fig. S1). This finding aligns with earlier studies demonstrating the antibacterial properties of *Anchusa* species. Specifically, *A. azurea* has been reported to inhibit the growth of 11 bacterial strains, including *E. coli*,* S. aureus* and with a less pronounced activity against *K. pneumoniae*. Moreover, *A. azurea* extracts exhibited moderate antibacterial activity against the standard strain, *A. baumannii* ATCC 19,606^62^. However, it is worth noting that ATCC 19,606 is susceptible to a wide range of antibiotics^63^, unlike the highly resistant *A. baumannii* AB5075, the strain used in this study. Overall, the antimicrobial results indicate a selective and comparatively modest antibacterial effect of AAME, in contrast to its more pronounced anticancer activity.

###  Determination of the phenolics and flavonoids contents

Phenolics and flavonoids are generally polar to moderately polar compounds; therefore, methanol facilitates their solubilization. Methanol has high polarity and well-documented efficiency in extraction of phenolic and flavonoids. Polyphenols and flavonoids content were obtained from the linear regression equation of the gallic acid and rutin calibration curves respectively^64^. The TPC and TFC of AAME was 5.46 mgGAE/gE and 0.13 mgRE/gE respectively. However, previous study reported higher values of TPC and TFC of the methanol extract (35.54 ± 0.4 mg GAE/gE and 1.88 ± 0.4 mgEQ/gE respectively)^65^. These results indicate that the methanol extract contains a considerable amount of flavonoids. The variation in TPC and TFC amounts can be attributed to many factors such as the plant species, extraction method, and solvent used.

HPLC method shows good accuracy and repeatability with recoveries of 98.13 and 98.87% respectively. Linearity was assessed by analysis of quercetin standard with average correlation coefficient of 0.9976. HPLC analysis of the AAME revealed the presence of 19 compounds with variant concentrations. The major identified phenolics were caffeic and chlorogenic acids (17020.93 ± 38.2 and 12315.2 ± 29.6 *µ*g/gE respectively). Rutin and hesperetin were the major identified flavonoids (8131.94 and 2509.22 *µ*g/gE, respectively) (Table 2). The results could confirm the probable rationale of the antioxidant and antimicrobial activities.

**Table 2:** HPLC analysis of the methanol extract of *A. azurea* aerial parts.

#	Rt	Metabolites	Conc. (µg/gE)
1	3.36	Gallic acid	507.76 ± 3.4
2	4.22	Chlorogenic acid	12315.2 ± 29.6
3	4.64	Catechin	476.72 ± 6.9
4	5.70	Methyl gallate	10499.1 ± 51.3
5	6.07	Caffeic acid	17020.93 ± 38.2
6	6.57	Syringic acid	1989.9 ± 36.7
7	7.12	Pyro catechol	80.92 ± 1.3
8	8.00	Rutin	8131.94 ± 21.8
9	8.49	Ellagic acid	1976.94 ± 23.5
10	9.18	Coumaric acid	827.86 ± 2.7
11	10.07	Ferulic acid	1338.16 ± 2.1
12	10.37	Naringenin	722.83 ± 2.3
13	12.07	Rosmarinic acid	204.66 ± 0.9
14	12.37	Daidzein	701.77 ± 2.6
15	12.71	Quercetin	1366.31 ± 1.7
16	13.95	Cinnamic acid	1501.48 ± 0.6
17	14.68	Apigenin	1831.54 ± 1.1
18	20.58	Kaempferol	378.1 ± 0.8
19	21.20	Hesperetin	2509.22 ± 2.4

Conc. (*µ*g/gE) indicates the mean ± S.E in triplicates.

### Identification of metabolomic profile of AAME by HRLC-MS/MS

**Fig. 10:**
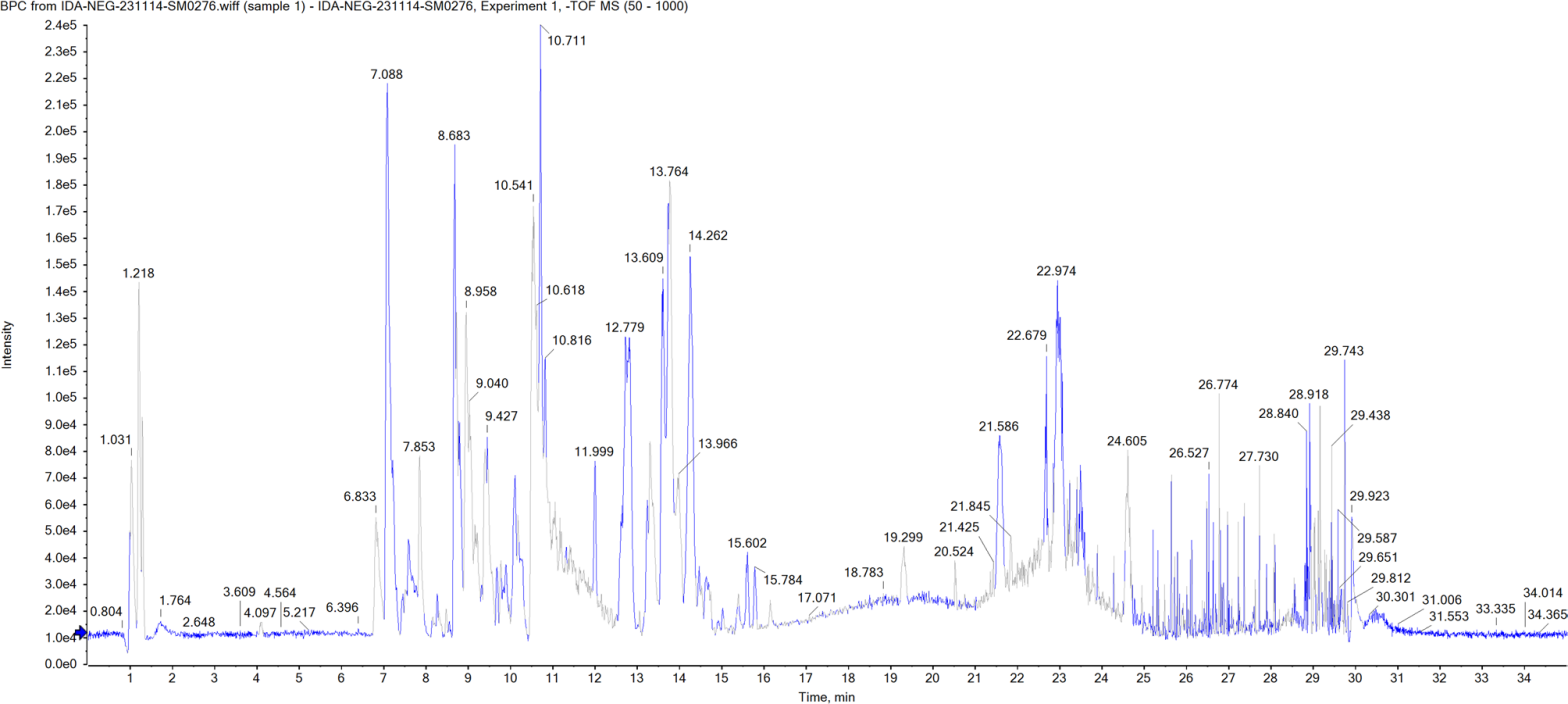
Base peak chromatogram of *A. azurea* methanol extract in HRLC-MS/MS negative ionization mode.

Metabolite identifications were established through the comparison of retention time and HR-MS/MS data, whenever possible, or by analyzing MS data in conjunction with literature findings. Various flavonoid aglycones along with their glycosides or glucuronic derivatives were recognized, notably with a prevalence of kaempferol and quercetin derivatives, alongside phenolic acids (Table 3; Fig. 10, Suppl. Fig. S2-S29). Polyphenols and flavonoids have garnered significant interest due to their advantageous impacts on health, including their antioxidant and antimicrobial properties^66^. *A. azurea* extract contains flavonol glycosides, with kaempferol diglycoside derivatives (M#1 and M#7) and kaempferol monoglycoside derivatives (M#3, M#8, M#12 and M#14), in addition to kaempferol aglycone (M#24) (Table 3). Kaempferol-di-*O*-hexoside (M#1) showed a deprotonated molecular ion peak at *m/z* 609.1395, and fragments at *m/z* 477, 285 signifies subsequent loss of 2 hexosyl moieties. Kaempferol-*O*-hexuronide (M#3, Suppl. Fig. S3) showed a deprotonated molecular ion peak at *m/z* 461.0896, and a fragment at *m/z* 285 signifies kaempferol aglycone, moreover its characteristic fragments (*m/z* 257 and 161). Kaempferol aglycone (M#24) was detected at *m/z* 285.0404 with its daughter peaks^67^. Quercetin-*O*-hexuronide (M#2, Suppl. Fig. S2) was detected by its parent ion at *m/z* 477.0692 [M-H] ^**−**^ and a fragment at *m/z* 301 for losing of hexuronyl moiety (176 Da), besides the characteristic fragments of quercetin aglycone *m/z* 179 and 151 for ^1,4^B^**−**^ and ^1,3^A^**−**^, respectively. The molecular ion peaks [M-H]^−^ of quercetin-*O*-hexoside (M#10, Suppl. Fig. S9) and quercetin-*O*-pentoside (M#11) were noticed at *m/z* 463.0849 and 433.0692, besides the characteristic fragments of the aglycone^67^. The expected losses of 18, 44, and 62 Da, equivalent to the loss of H_2_O, CO_2_, and both, respectively, were observed in MS2 fragmentations of acids as in coumaric and ferulic acids (M#29, M#30, Suppl. Fig. S20, 21). The proposed method was useful for the characterization of rosmarinic acid (M#37).

**Table 3:** Tentatively identified metabolites in *A. azurea* methanol extract *via* HRLC-T-TOF-MS/MS using negative ionization mode.

M#	Rt	Metabolites	Formula	M-H	Error (ppm)	MS^2^
Flavonoids						
1	7.29	Kaempferol-di-*O*-hexoside	C_27_H_30_O_16_	609.1395	8.7	477, 285
2	7.51	Quercetin-*O*-hexuronide	C_21_H_18_O_13_	477.0692	5.2	301, 179, 151
3	7.68	Kaempferol-*O*-hexuronide	C_21_H_18_O_12_	461.0896	17.7	285, 257, 161
4	7.83	Acacetin-*O*-pentoside	C_21_H_20_O_9_	415.1225	9.4	283, 252, 153
5	8.24	Rutin	C_27_H_30_O_16_	609.1441	-3.3	301
6	8.38	Apigenin-*C*-hexoside	C_21_H_20_O_10_	431.1017	7.6	341, 311, 269, 179, 161
7	8.63	Kaempferol-*O*-neohesperidoside	C_27_H_30_O_15_	593.1505	1.1	447, 285
8	8.63	Kaempferol-*O*-coumaroyl-hexoside	C_30_H_26_O_13_	593.1472	19.7	285, 255, 151
9	8.84	Isorhamnetin-*O*-rutinoside	C_28_H_32_O_16_	623.1712	15.1	461, 315
10	8.86	Quercetin-*O*-hexoside	C_21_H_20_O_12_	463.0849	-6.2	301, 151
11	8.86	Quercetin-*O*-pentoside	C_20_H_18_O_11_	433.0692	-18.3	301, 225, 179, 161, 135
12	8.88	Kaempferol-*O*-hexoside	C_21_H_20_O_11_	447.0927	-1.3	285, 227, 151
13	8.97	Isorhamnetin-*O*-hexoside	C_22_H_22_O_12_	477.0983	-10.6	315, 179, 161
14	9.03	Kaempferol-*O*-pentoside	C_20_H_18_O_10_	417.0708	-13.9	285
15	9.07	Quercetin	C_16_H_14_O_6_	301.0255	-18.1	287, 271, 255, 179, 161
16	9.41	Phlorizin	C_21_H_24_O_10_	435.1210	-20.0	273, 167
17	9.58	Apigenin-*O*-hexoside	C_21_H_20_O_10_	431.0938	-10.7	269, 255, 161
18	9.63	Naringenin-*O*-hexoside	C_21_H_22_O_10_	433.1152	2.8	271, 161, 133
19	11.18	Naringenin	C_15_H_12_O_5_	271.0634	11.4	177, 151, 119, 107
20	11.59	Apigenin	C_15_H_10_O_5_	269.0461	14.3	225, 151, 117
21	11.79	Catechin	C_15_H_14_O_6_	289.0763	15.7	271, 181
22	12.22	Acacetin	C_16_H_12_O_5_	283.0663	19.2	268, 161, 151
23	13.04	Kaempferide	C_16_H_12_O_6_	299.0583	9.0	213, 137
24	15.09	Kaempferol	C_15_H_10_O_6_	285.0404	1.4	257, 177, 133
25	20.56	Isorhamnetin	C_16_H_12_O_7_	315.0539	10.6	271, 151
Phenolic acids						
26	1.12	Dihydroxybenzoic acid	C_7_H_6_O_4_	153.0191	-1.3	109, 91
27	1.44	Chlorogenic acid	C_16_H_18_O_9_	353.0858	-5.7	191, 179, 135
28	2.01	Caffeic Acid	C_9_H_8_O_4_	179.0355	2.8	161, 143, 135, 99
29	3.05	Coumaric acid	C_9_H_8_O_3_	163.0420	11.7	145, 119
30	4.17	Ferulic acid	C_10_H_10_O_4_	193.0504	1.6	178, 161, 149, 133
31	4.59	Hydroxybenzoic acid	C_7_H_6_O_3_	137.0240	-2.9	93
32	5.15	Homogenentisic acid	C_8_H_8_O_4_	167.0338	-7.2	149, 123, 108
33	6.20	Sinapic acid	C_11_H_12_O_5_	223.0632	9	193, 179
34	6.34	Hydroxyphenylacetic acid	C_8_H_8_O_3_	151.0379	-14.6	133, 107
35	6.43	Dihydroxymandelate	C_8_H_8_O_5_	183.0354	19.7	165, 139
36	6.77	Quinic acid	C_7_H_12_O_6_	191.0560	-0.5	173, 127, 93
37	7.09	Rosmarinic acid	C_18_H_16_O_8_	359.0767	1.4	-
38	7.45	Shikimic acid	C_7_H_10_O_5_	173.0434	-9.2	155, 137, 93
39	8.91	Methoxysalicylic acid	C_8_H_8_O_4_	167.0348	-1.2	152, 123, 108
40	10.44	Methyl salicylate	C_8_H_8_O_3_	151.0397	-2.6	136, 92
Acids						
41	0.90	Maleic acid	C_4_H_4_O_4_	115.0027	-8.7	97, 71
42	0.92	Succinic acid	C_4_H_6_O_4_	117.0184	-5.1	99, 73
43	0.98	Malic acid	C_4_H_6_O_5_	133.0146	3	115, 89, 71
44	1.02	Lactic acid	C_3_H_6_O_3_	89.02398	-4.7	71
45	1.02	Tartaric acid	C_4_H_6_O_6_	149.0073	-10.7	105
46	1.13	Hydroxybutyric acid	C_4_H_8_O_3_	103.0419	17.5	85
47	1.22	Suberic acid	C_8_H_14_O_4_	173.0805	-10.4	129
48	1.60	Isopropylmalic acid	C_7_H_12_O_5_	175.0632	11.4	131
49	19.30	Linolenic acid	C_18_H_30_O_2_	277.2176	1.1	259, 233
50	22.66	Hydroxypalmetic acid	C_16_H_32_O_3_	271.2307	10.3	253, 225

### Radical scavenging assay

AAME showed low radical scavenging activity with an EC_50_ value of 209.67 ± 1.58 *µ*g/mL compared to gallic acid and rutin (42.36 ± 0.58 and 13.24 ± 0.48 *µ*g/mL respectively). The relatively higher EC₅₀ value indicates a lower antioxidant potency, which is consistent with the low TPC and TFC observed in the extract. The presence of identified phenolic acids, such as caffeic, chlorogenic, and *p*-hydroxybenzoic acids, may contribute to the observed radical scavenging activity. However, the overall moderate activity reflects the limited abundance of these bioactive compounds in the extract.

## Conclusion

The combination of *A. azurea* methanol extract with Cisplatin demonstrated in vitro synergistic anticancer effects, associated with the downregulation of key pro-inflammatory and survival pathways, including TNF-α, IL-17, NF-κB, JNK, and MAPK. These effects were accompanied by enhanced caspase activation, induction of autophagy, and cell cycle arrest, suggesting pro-apoptotic and anti-proliferative activity. Major identified metabolites, including kaempferol, quercetin, rosmarinic acid, and α-linolenic acid, may contribute to these effects, potentially through modulation of TNF-α signaling. Network pharmacology analysis further supported the relevance of these compounds by identifying shared targets associated with oral cancer, indicating that *A. azurea* may act as a multi-target modulator of inflammatory and apoptotic pathways.

However, these findings are based on in vitro models, and therefore, their translational relevance should be interpreted with caution. Further in vivo studies are essential to validate the efficacy, safety, and pharmacokinetic interactions of *A. azurea* in combination with cisplatin. In addition, future investigations focusing on the functional validation of the implicated molecular pathways, particularly TNF-α and IL-17 signaling, will provide deeper mechanistic insights. Expanding the evaluation to include additional normal cell models (e.g., keratinocytes or oral fibroblasts) would further strengthen the assessment of tissue specificity and safety.

## Supplementary Information

Below is the link to the electronic supplementary material.

Supplementary Material 1

Supplementary Material 2

Supplementary Material 3

Supplementary Material 4

## Data Availability

Data will be made available upon request.

## Abbreviations

AAME: *Anchusa azurea* methanol extract
AO: Acridine orange
CI: Combination index
Cis: Cisplatin
DMSO: Dimethyl sulfoxide
DRI: Dose reduction index
NFI: Net fluorescent intensities
OD: Optical density
PBS: Phosphate buffered saline
SRB: Sulphorhodamine-B
TSA: Tryptic Soy Agar
