# Supplementary material for: Anchusa azurea enhances cisplatin efficacy in oral and bone cancers through IL-17 and TNF-α pathway modulation: a metabolomic and network pharmacology approach
Source: Sci Rep. 2026 Jun 13;16:18366. doi: 10.1038/s41598-026-56489-3 (PMC13264614; doi:10.1038/s41598-026-56489-3)
Supplement: Supplementary file 1 — Supplementary Material 1 [file 41598_2026_56489_MOESM1_ESM.pdf]

Table S1. IC50 values of AAME and Cis on various cell lines

**Treatment**	**Cell line**	**IC50 (µg/mL)**
**AAME**	MG-63 (bone)	156.2
	HNO97 (oral)	139.2
	A-375 (melanoma)	>500*
	HSF (normal fibroblasts)	>500*
**Cisplatin (Cis)**	HSF (normal fibroblasts)	24.53
	MG-63 (bone)	16.87
	HNO97 (oral)	7.045
	A-375 (melanoma)	4.85

Table S2: Combination and dose reduction indices of A.Azurea and Cis combinations

**HNO97**					**MG63**				
**A. Azurea (ug/ml)**	**Cis (ug/ml)**	**CI**	**DRI A.Azurea**	**DRI Cis**	**A. Azurea (ug/ml)**	**Cis (ug/ml)**	**CI**	**DRI A.Azurea**	**DRI Cis**
**15.625**		0.098	107.02	11.28	**15.62**		0.29	35.77	3.75
**31.25**		0.087	65.75	13.82	**31.25**		0.18	31.29	6.57
**62.5**	**7.045**	0.079	42.59	17.84	**62.5**		0.15	22.36	9.39
**125**		0.058	37.35	31.06	**125**	**16.82**	0.19	11.12	9.35
**250**		0.055	28.77	47.59	**250**		0.27	5.87	9.87
**500**		0.066	19.57	64.50	**500**		0.29	4.34	14.58
**15.625**		0.201	28.18	6.041	**15.62**		0.74	7.76	1.62
**31.25**		0.194	17.17	7.35	**31.25**		0.55	6.17	2.58
**62.5**		0.157	13.84	11.77	**62.5**		0.48	4.51	3.78
**125**	**3.5225**	0.189	8.4	14.25	**125**	**8.41**	0.28	5.69	9.57
**250**		0.107	12.13	40.59	**250**		0.34	3.78	12.71
**500**		0.129	8.914	59.35	**500**		0.32	3.56	23.98

CI: combination index, DRI: dose reduction index

Table S3. Gene Ontology Enrichment Analysis

FDR	nGenes	Pathway Genes	Fold Enrichment	Pathway	Genes
**1.41E-14**	10	41	62.01	Path:hsa05219 Bladder cancer	DAPK1 EGFR ERBB2 MDM2 MMP1 MMP2 MMP9 MAPK1 SRC BRAF
**2.19E-16**	13	79	41.83	Path:hsa01521 EGFR tyrosine kinase inhibitor resistance	EGFR ERBB2 AKT1 GSK3B IGF1R IL6 KDR MET PIK3R1 AXL MAPK1 SRC BRAF
**9.09E-17**	14	95	37.46	Path:hsa01522 Endocrine resistance	MAPK14 EGFR ERBB2 AKT1 ESR1 IGF1R MDM2 MMP2 MMP9 PIK3R1 MAPK1 MAPK8 SRC BRAF
**3.03E-12**	10	72	35.31	Path:hsa05223 Non-small cell lung cancer	CDK6 EGFR ERBB2 AKT1 MET PIK3R1 MAPK1 RARB RXRA BRAF
**3.04E-15**	13	97	34.07	Path:hsa05215 Prostate cancer	CDK2 EGFR ERBB2 AKT1 GSK3B IGF1R AR MDM2 MMP3 MMP9 PIK3R1 MAPK1 BRAF
**4.56E-14**	12	93	32.8	Path:hsa04657 IL-17 signaling pathway	MAPK14 GSK3B IL6 MMP1 MMP3 MMP9 MMP13 MAPK1 MAPK8 PTGS2 CASP3 CASP8
**6.70E-15**	13	104	31.78	Path:hsa04625 C-type lectin receptor signaling pathway	MAPK14 AKT1 IL2 IL6 MDM2 PIK3R1 MAPK1 MAPK8 PTGS2 SRC SYK CASP1 CASP8
**1.41E-14**	13	112	29.51	Path:hsa04668 TNF signaling pathway	MAPK14 AKT1 IL6 MMP3 MMP9 MMP14 PIK3R1 MAPK1 MAPK8 PTGS2 CASP3 CASP7 CASP8
**3.04E-12**	11	102	27.42	Path:hsa04914 Progesterone-mediated oocyte maturation	CDK2 MAPK14 AKT1 IGF1R PIK3R1 PLK1 MAPK1 MAPK8 BRAF CDK1 CDC25A
**1.69E-12**	12	129	23.65	Path:hsa04926 Relaxin signaling pathway	MAPK14 EGFR AKT1 MMP1 MMP2 MMP9 MMP13 NOS2 PIK3R1 MAPK1 MAPK8 SRC
**1.92E-12**	12	131	23.29	Path:hsa04068 FoxO signaling pathway	CDK2 MAPK14 EGFR AKT1 IGF1R IL6 MDM2 PIK3R1 PLK1 MAPK1 MAPK8 BRAF
**3.64E-13**	13	148	22.33	Path:hsa05226 Gastric cancer	CDK2 EGFR ERBB2 AKT1 GSK3B MET ABCB1 PIK3R1 MAPK1 RARB RXRA BRAF TERT
**4.56E-14**	14	161	22.11	Path:hsa05206 MicroRNAs in cancer	CDK6 CYP1B1 EGFR ERBB2 MDM2 MET MMP9 ABCB1 PIM1 PIK3R1 MAPK1 PTGS2 CASP3 CDC25A
**1.89E-16**	17	202	21.39	Path:hsa05205 Proteoglycans in cancer	MAPK14 EGFR ERBB2 AKT1 ESR1 IGF1R KDR MDM2 MET MMP2 MMP9 PIK3R1 MAPK1 PTPN6 SRC BRAF CASP3
**3.57E-17**	18	214	21.38	Path:hsa05417 Lipid and atherosclerosis	MAPK14 AKT1 GSK3B IL6 MMP1 MMP3 MMP9 PIK3R1 PPARG MAPK1 MAPK8 RXRA SRC CASP1 CASP3 CASP6 CASP7 CASP8
**4.96E-13**	14	194	18.35	Path:hsa05167 Kaposi sarcoma-associated herpesvirus infection	CDK6 MAPK14 AKT1 GSK3B IL6 PIK3CG PIK3R1 MAPK1 MAPK8 PTGS2 SRC SYK CASP3 CASP8
**1.71E-34**	37	530	17.75	Path:hsa05200 Pathways in cancer	CDK2 CDK6 DAPK1 EDNRA EGFR ERBB2 AKT1 ESR1 F2 FLT3 GSK3B IGF1R IL2 IL6 AR MDM2 MET MMP1 MMP2 MMP9 NOS2 PIM1 PIK3R1 PPARG MAPK1 MAPK8 PTGER1 PTGER4 PTGS2 RARA RARB RXRA BRAF TERT CASP3 CASP7 CASP8
**1.96E-13**	15	224	17.02	Path:hsa05163 Human cytomegalovirus infection	CDK6 MAPK14 EGFR AKT1 GSK3B IL6 MDM2 PIK3R1 MAPK1 PTGER1 PTGER4 PTGS2 SRC CASP3 CASP8
**4.38E-12**	14	232	15.34	Path:hsa05171 Coronavirus disease-COVID-19	MAPK14 ACE EGFR F2 IL2 IL6 MMP1 MMP3 PIK3R1 MAPK1 MAPK8 SYK ADAM17 CASP1
**4.56E-14**	18	354	12.93	Path:hsa04151 PI3K-Akt signaling pathway	CDK2 CDK6 EGFR ERBB2 AKT1 FLT3 GSK3B IGF1R IL2 IL6 KDR MDM2 MET PIK3CG PIK3R1 MAPK1 RXRA SYK
