# Supplementary material for: Anchusa azurea enhances cisplatin efficacy in oral and bone cancers through IL-17 and TNF-α pathway modulation: a metabolomic and network pharmacology approach
Source: Sci Rep. 2026 Jun 13;16:18366. doi: 10.1038/s41598-026-56489-3 (PMC13264614; doi:10.1038/s41598-026-56489-3)
Supplement: Supplementary file 3 — Supplementary Material 3 [file 41598_2026_56489_MOESM3_ESM.pdf]

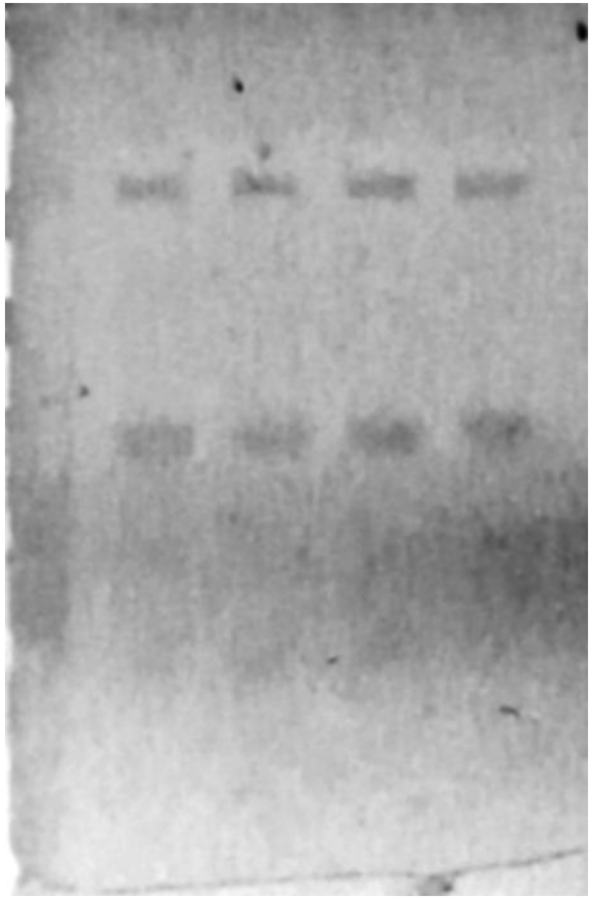

NFκB/65 KDa

β actin/43 KDa

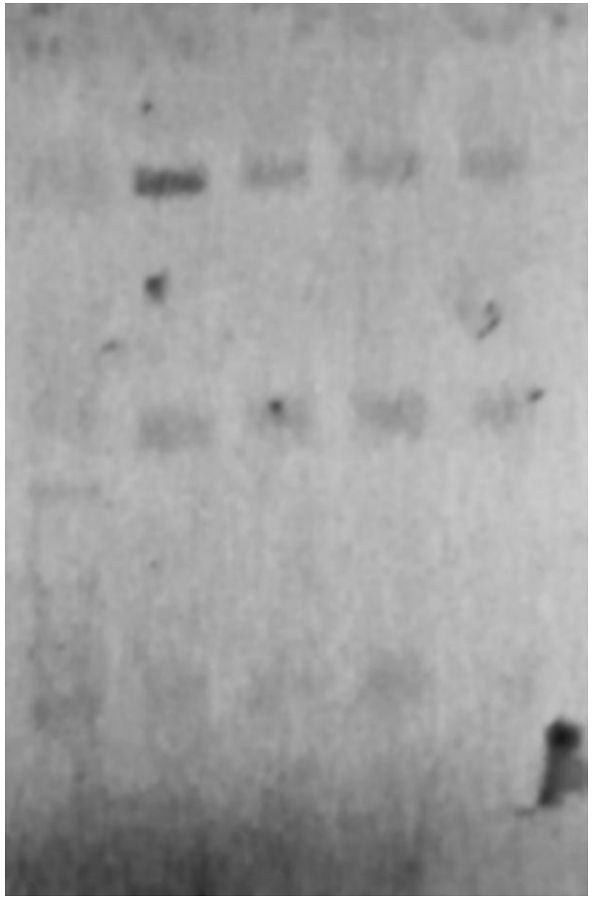

p-NFκB/65 KDa

β actin/43 KDa

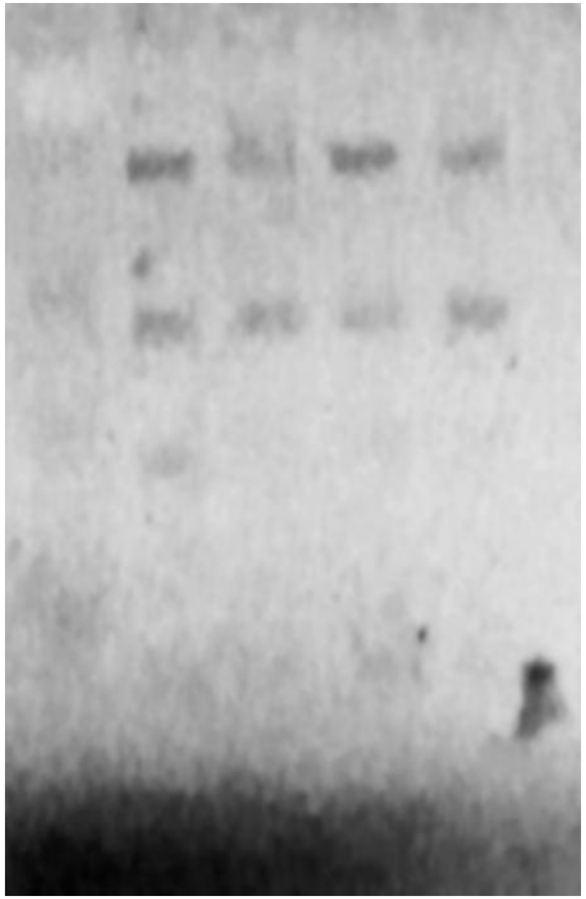
p-JNK/54 KDa
p-JNK/46 KDa

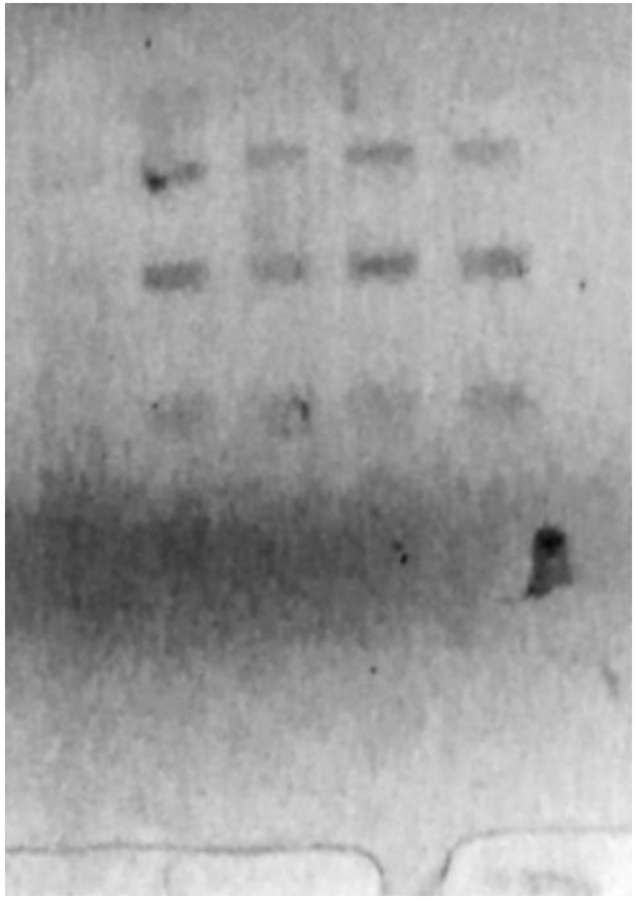
JNK/54 KDa
JNK/46 KDa

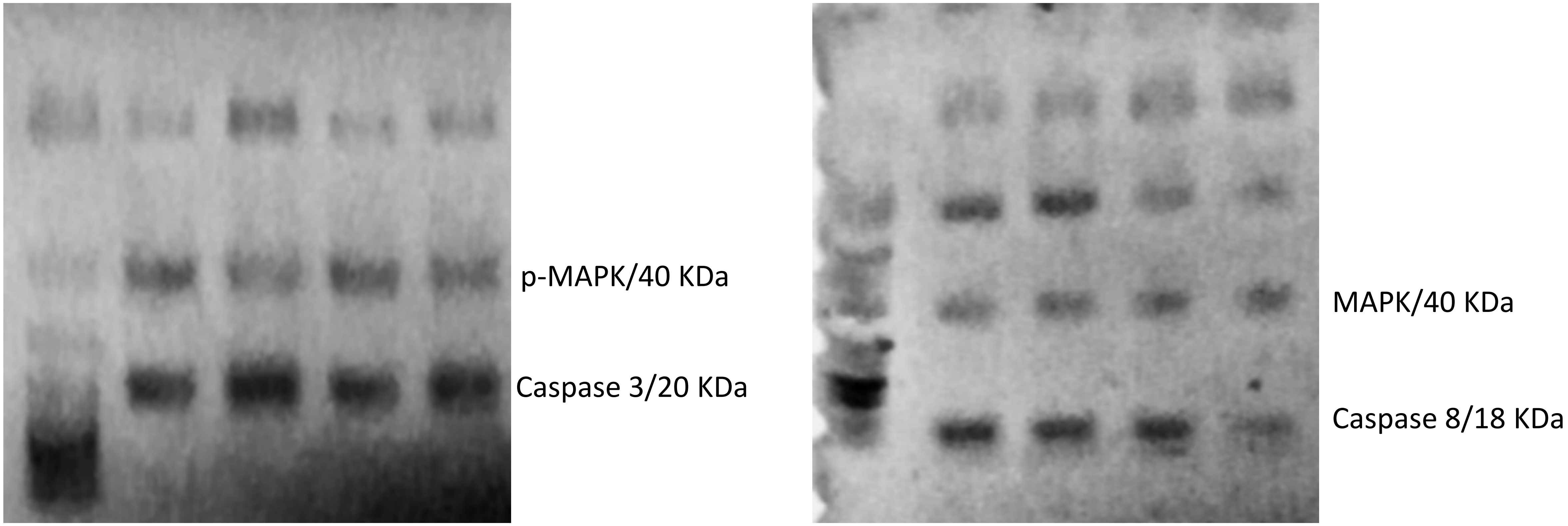
p-MAPK/40 KDa
Caspase 3/20 KDa
MAPK/40 KDa
Caspase 8/18 KDa

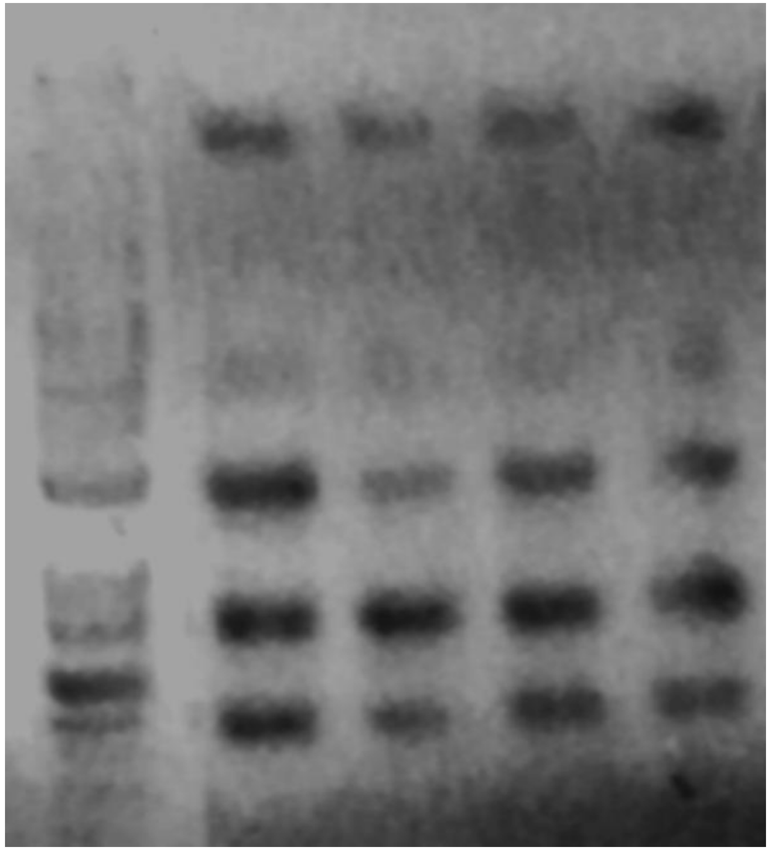

TRAF/60 KDa

AP1/36 KDa

TNFα/20 KDa

IL-17/15 KDa
