# Supplementary figures and images for: Anchusa azurea enhances cisplatin efficacy in oral and bone cancers through IL-17 and TNF-α pathway modulation: a metabolomic and network pharmacology approach
Source: Sci Rep. 2026 Jun 13;16:18366. doi: 10.1038/s41598-026-56489-3 (PMC13264614; doi:10.1038/s41598-026-56489-3)

Uneditted blots

AP1


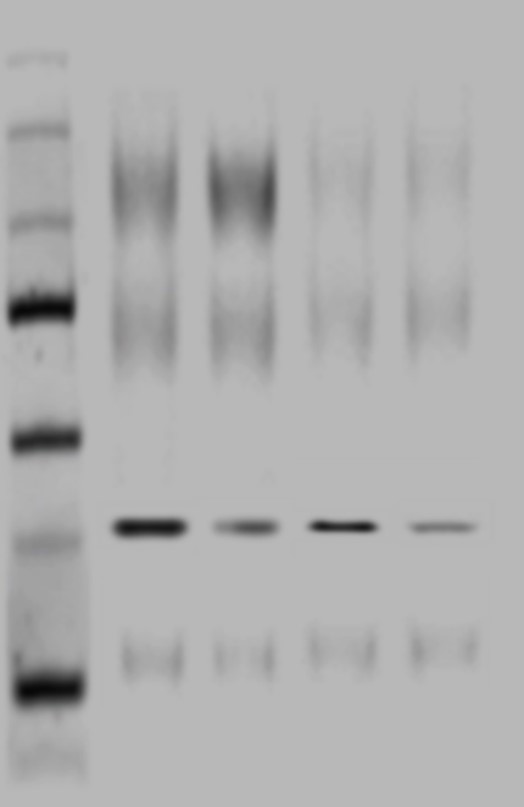


Caspase 3


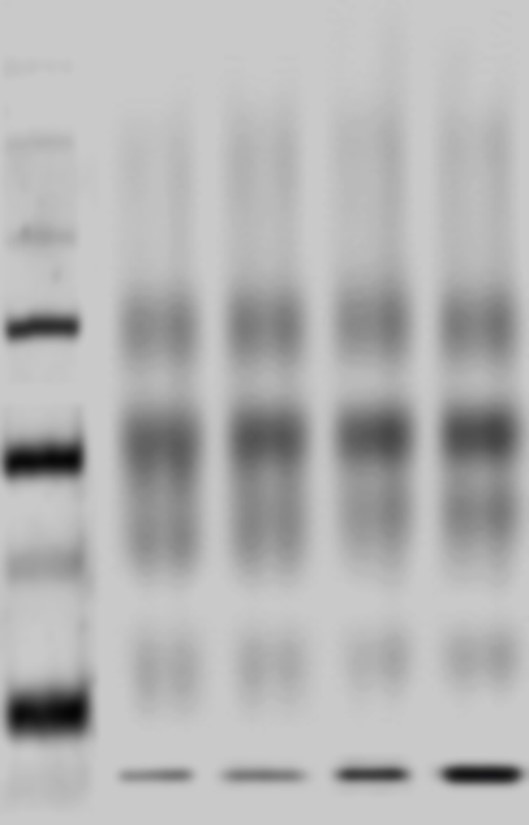


Caspase 8


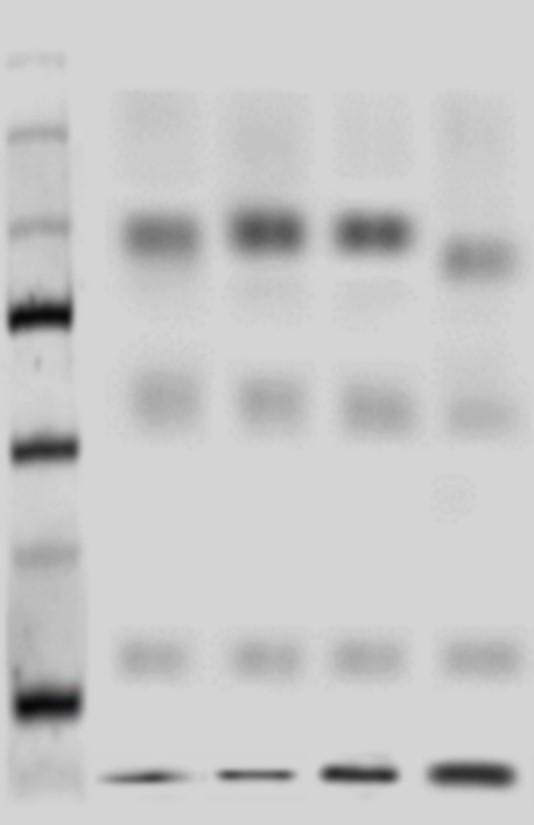


IL-17


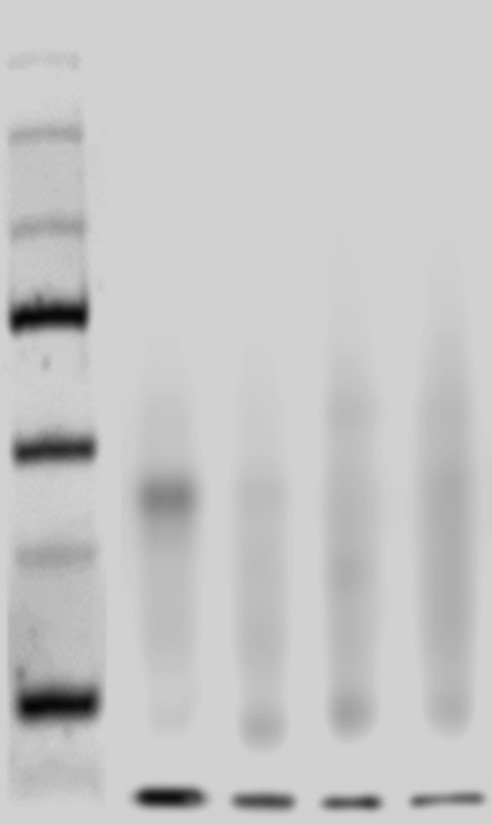


JNK


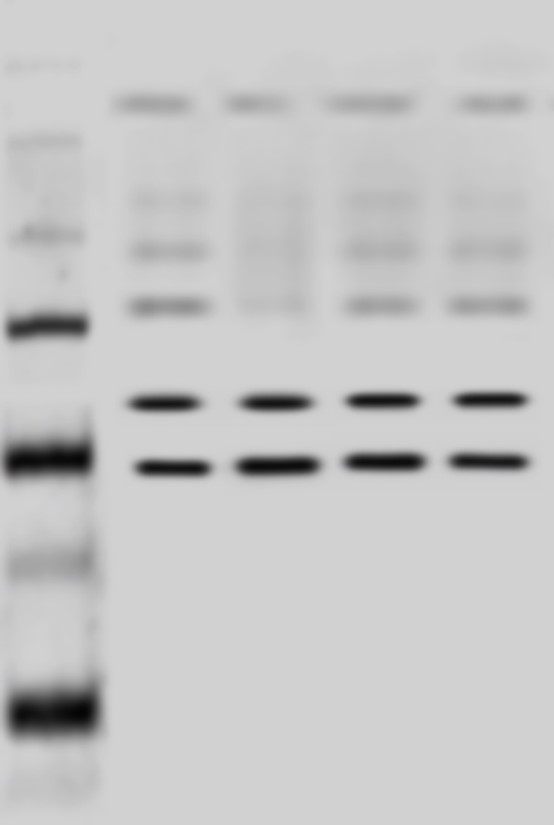


pJNK


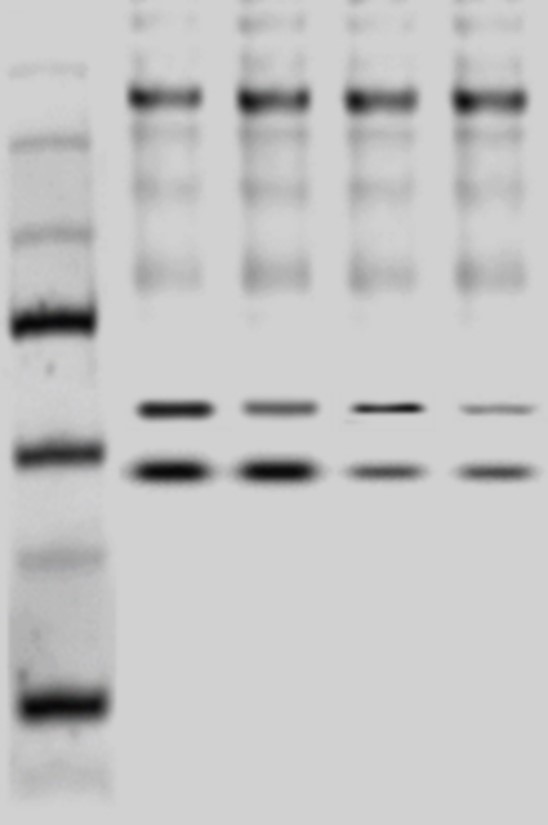


NFKB


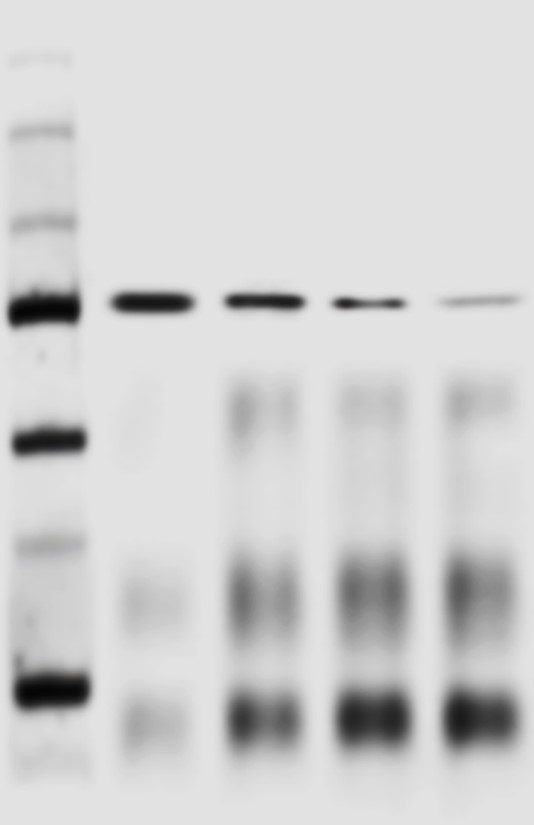


p-NFKB


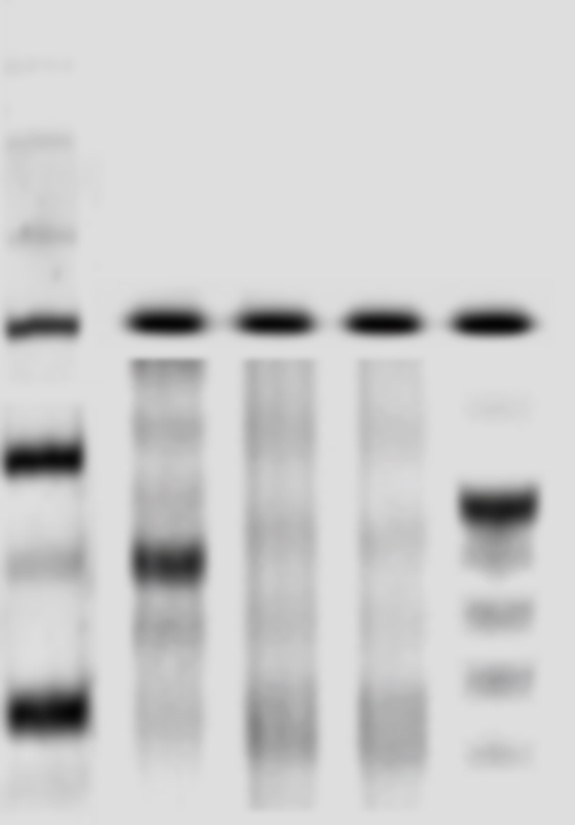


P38 MAPK


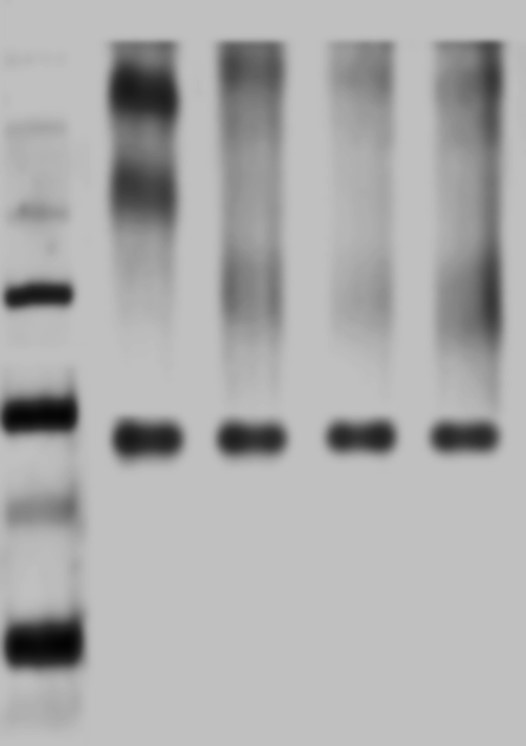


p-p38 MAPK


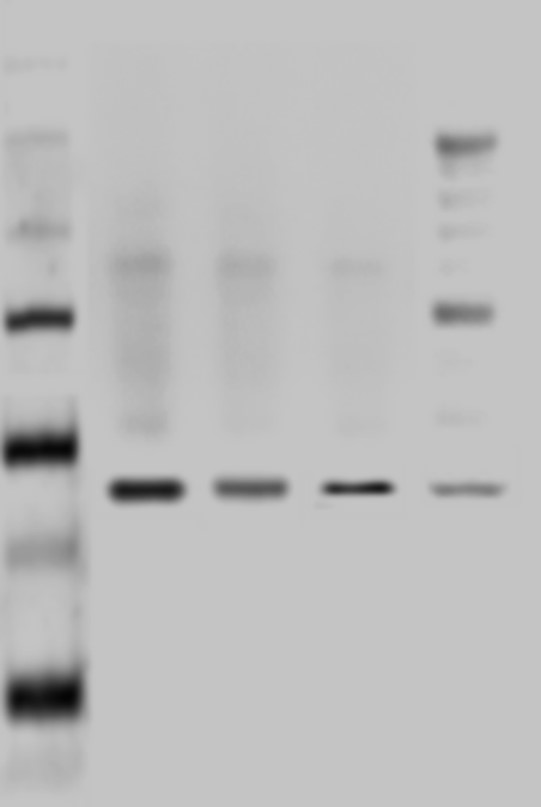


TNFalpha


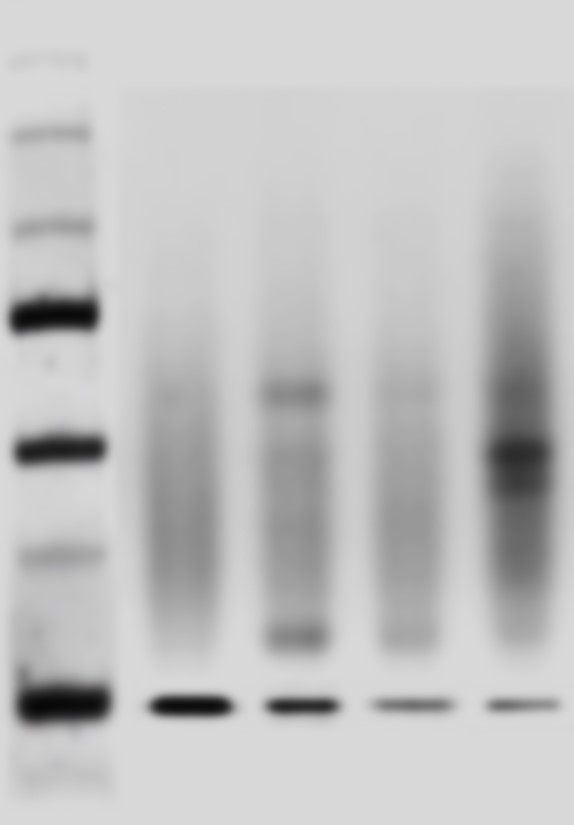


TRAF6


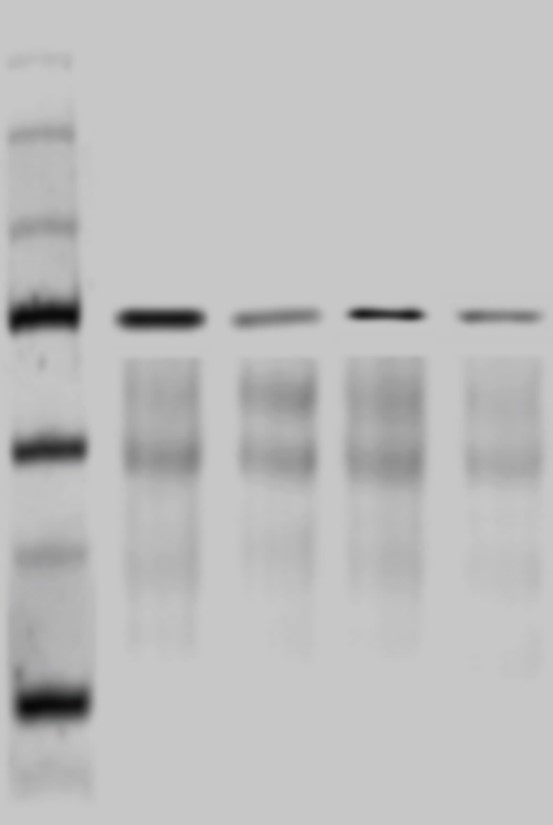

Supplement: Supplementary file 4 — Supplementary Material 4 [file 41598_2026_56489_MOESM4_ESM.docx]
